# In Situ Valence Engineering of Copper Silicate Nanozymes with Enhanced Peroxidase‐Like Catalytic Activity for Oral Disease Detection

**DOI:** 10.1002/advs.202503237

**Published:** 2025-06-05

**Authors:** Xiaocan Liu, Zhen Ding, Chengjing Xu, Jinming Zhang, Yufu Liu, Tianyan Chen, Shuang Dai, Xingfu Bao, Min Hu, Zhen Liu

**Affiliations:** ^1^ Jilin Provincial Key Laboratory of Tooth Development and Bone Remodeling School and Hospital of Stomatology Jilin University Changchun 130021 China; ^2^ Key Laboratory of Pathobiology Ministry of Education Jilin University Changchun 130021 China; ^3^ Beijing Advanced Innovation Center for Soft Matter Science and Engineering College of Life Science and Technology Beijing University of Chemical Technology Beijing 100029 China

**Keywords:** nanozymes, periodontitis, peroxidase‐like catalytic activity, valence engineering, volatile sulfur compounds (VSCs)

## Abstract

As a series of attractive nanomaterials, nanozymes with great catalytic activity and specificity are well developed in the field of biosensors. Although promising, the lack of appropriate structural design strategy and limitation of sensing performance in the clinical samples remain challenging for the practical application of nanozymes. Herein, a novel copper silicate nanozyme (CSHSs‐Ar) with enhanced peroxidase‐like catalytic activity is synthesized through a facile in situ valence‐engineered approach. After the optimization of synthesis, the resultant CSHSs‐Ar nanozymes containing nearly 60% of Cu^+^ hold a higher peroxidase‐like catalytic activity and a better catalytic specificity than the other two derivatives (CSHSs and CSHSs‐air). Theoretical calculations also demonstrate that CSHSs‐Ar nanozymes are more beneficial toward the activation of H_2_O_2_ compared with CSHSs and CSHSs‐air. On this basis, the well‐developed CSHSs‐Ar nanozyme‐involved system is employed as an efficient colorimetric sensor for the detection of volatile sulfur compounds (VSCs) and prediction for periodontitis. Moreover, several visual molecular logic gates are explored as a proof of concept to the application of CSHSs‐Ar nanozymes with superior peroxidase‐like catalytic activity. This study not only provides guidance for the development of novel nanozymes, but also broadens the biomedical application potential of nanozymes including the detection of oral diseases.

## Graphical abstract

A novel copper silicate nanozyme (CSHSs‐Ar) with high peroxidase‐like catalytic activity and great catalytic specificity is well developed using a facile in situ valence‐engineered approach. The well‐developed CSHSs‐Ar nanozyme‐involved system can be utilized and employed as an efficient colorimetric sensor for the detection of volatile sulfur compounds (VSCs) and prediction for the clinical oral diseases with different degrees.

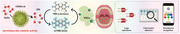

## 1 Introduction

As a universal oral disease worldwide with an over 30% incidence in adults, periodontitis has seriously affected the mastication, self‐esteem, and social activity of patients due to tooth loss, gingival recession, and halitosis.^[^ ^1^ ^]^ In addition to the impact on oral health, there is a close correlation between periodontitis and some systemic diseases including diabetes, Alzheimer's disease, rheumatoid arthritis, and renal injury.^[^ ^2^ ^]^ On this basis, early diagnosis of periodontitis can significantly reduce the symptoms of oral disease and even the risk of tooth loss.^[^ ^3^ ^]^ However, the diagnosis of early‐stage periodontitis highly relies on the clinical experience of dentists.^[^ ^4^ ^]^ To achieve daily oral health management, it is urgently needed to develop a convenient and low‐cost approach for early screening and monitoring periodontitis. Over the past decades, the occurrence and development of periodontitis mainly originates from the ecological succession of resident microbiota.^[^ ^5^ ^]^ The oral microbiota of healthy individuals usually holds a balanced and dynamic ecosystem while the bacterial community may undergo profound changes during periodontitis.^[^ ^6^ ^]^ It is well known that the oral pathogens associated with periodontitis can produce a large amount of volatile sulfur compounds (VSCs) including H_2_S.^[^ ^7^ ^]^ As an indicator for the progression of periodontitis, H_2_S can well reflect the changes in microbial metabolism and oral microenvironment.^[^ ^8^ ^]^ Thus, it is possible to monitor the number of periodontal pathogens and the degree of periodontal disease by detecting the change of VSCs in oral cavity.

Because of their high stability, facile synthesis, and potential for large‐scale production, nanozymes with peroxidase (POD)‐like catalytic activity have served as efficient candidates for POD in biosensing, antitumor therapy, and antibacterial treatment.^[^ ^9^ ^]^ Meanwhile, colorimetric approaches based on POD‐like nanozymes have been well proposed for the visible detection of biothiols.^[^ ^10^ ^]^ Despite these advancements, the catalytic efficiency and specificity between natural POD and POD‐like nanozymes seriously limit their practical application and further development.^[^ ^11^ ^]^ To overcome these shortcomings, recent synthetic strategies in the design of efficient POD‐like nanozymes mainly concentrate on the reduction in size toward single‐atom or zero dimensions, modification in crystallinity or crystal defect, alteration of surface property with organic ligands or functional groups, and additional introduction of active sites or oxygen vacancies.^[^ ^12^ ^]^ Even so, these studies are restricted to the complicated synthesis, excessive use of organic reagents, and low production.^[^ ^13^ ^]^ Apart from the above‐mentioned approaches, rational regulation of the valence state distribution of metals in POD‐like nanozymes can create well‐defined catalytic sites by controlling structural and electronic effects.^[^ ^14^ ^]^ Moreover, highly ordered catalytic sites in POD‐like nanozymes can endow them with higher stability and further successfully drive the catalysis.^[^ ^15^ ^]^ Although promising, it is still a challenge to achieve efficient POD‐like nanozymes with well‐regulated valence state distribution and clear catalytic mechanisms upon a facile synthetic approach. More importantly, low valene metals in the resultant POD‐like nanozymes are highly susceptible to being oxidized into the high valence ones at times owing to their low redox potential.^[^ ^16^ ^]^ Accordingly, developing novel valence‐engineered POD‐like nanozymes with appropriate valence state distribution and understanding their formation mechanism and catalytic performance are of great significance.

Till now, a series of copper‐assisted Fenton‐like catalysts with admirable POD‐like catalytic activity have been reported for the chemodynamic therapy of tumors and wound infections. The common strategy of these studies against tumors mainly depends on the glutathione‐assisted Cu^2+^ reduction in tumor cells and the Cu^+^‐involved Fenton‐like reaction.^[^ ^17^ ^]^ Also, copper‐based POD‐like nanozymes with low‐valence Cu^+^ and Cu^0^ usually hold better outcomes than those with high‐valence Cu^2+^ in the chemodynamic therapy of wound infections.^[^ ^18^ ^]^ Considering the above‐mentioned factors, we envision that the design of copper‐based POD‐like nanozymes incorporating abundant low‐valence copper and well‐defined chemical structures may hold significant promise as a new platform for the advanced biosensing for clinical samples.

**Scheme 1:**
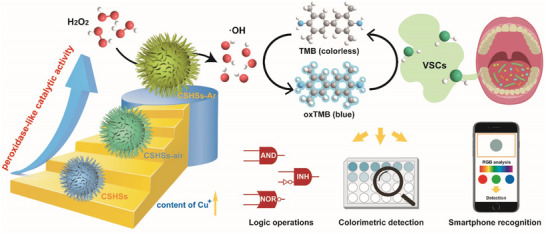
Schematic illustration of the POD‐like catalytic activity of CSHSs‐Ar and the application in the detection of VSCs and diagnosis of periodontitis.

Herein, we employed a facile in situ valence‐engineered strategy to fabricate a novel copper silicate nanozyme (CSHSs‐Ar) with superior POD‐like catalytic activity, and the resultant CSHSs‐Ar nanozyme was proposed for the detection of VSCs and prediction of periodontitis (**Scheme** **1**). Notably, the ≈60% Cu^+^ in CSHSs‐Ar nanozymes endowed them with a higher POD‐like catalytic activity and specificity than the other two derivatives (CSHSs and CSHSs‐air). Based on the theoretical analysis, CSHSs‐Ar nanozymes were more beneficial toward the activation of H_2_O_2_ compared with CSHSs and CSHSs‐air. Therefore, CSHSs‐Ar nanozymes were utilized to develop an efficient nanozyme‐linked colorimetric assay for the visible detection of VSCs. With the assistance of smartphone and color recognition software, we also constructed a paper‐based colorimetric sensing platform for VSCs toward the need of point‐of‐care testing. Moreover, CSHSs‐Ar nanozyme‐assisted detection could indicate the oral health condition by monitoring the VSCs generated from oral pathogens during their proliferation. Beyond this, several visual molecular logic gates were designed owing to the great sensing sensitivity and accuracy of CSHSs‐Ar nanozyme‐assisted detection platform. Accordingly, our study not only provided great prospects for valence engineering in the design of highly active POD‐like nanozymes, but also expanded the practical application of nanozymes in biosensing toward clinical needs.

## 2 Results and Discussion

### 2.1 Synthesis and Characterizations of CSHSs, CSHSs‐air, and CSHSs‐Ar

**Figure 1:**
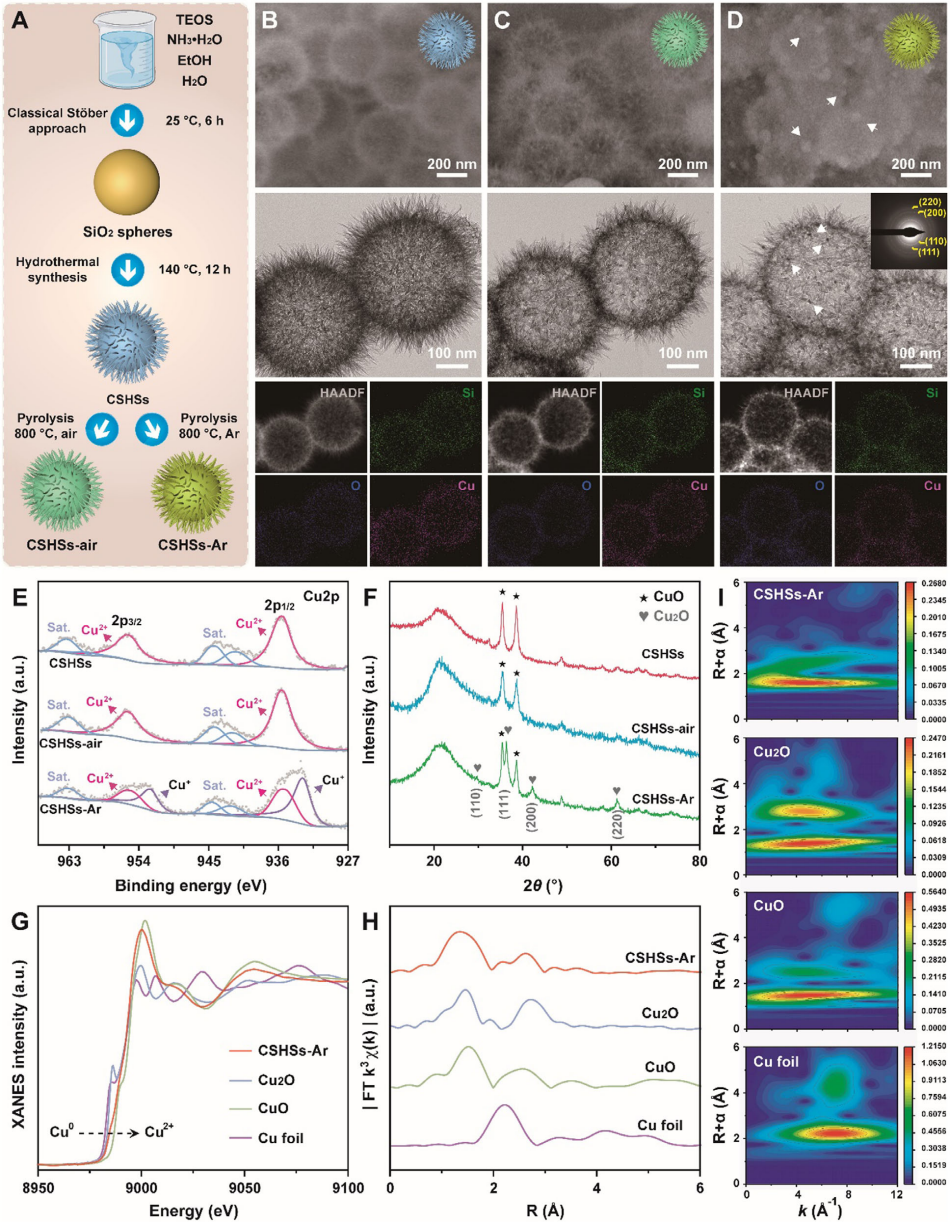
A) Schematic illustration of the synthesis of CSHSs, CSHSs‐air, and CSHSs‐Ar. SEM, TEM, and elemental mapping images of B) CSHSs, C) CSHSs‐air, and D) CSHSs‐Ar. E) Cu 2p XPS spectra and F) XRD patterns of CSHSs, CSHSs‐air, and CSHSs‐Ar. G) The Cu K‐edge XANES spectra of CSHSs‐Ar and their references including Cu foil, Cu_2_O, and CuO. H) Cu K‐edge FT k^3^‐weighted EXAFS spectra of CSHSs‐Ar and their references. I) The WT‐EXAFS profiles of CSHSs‐Ar and their references.

As illustrated in **Figure** **1**A, copper silicate hollow spheres (CSHSs) were first synthesized via a facile hydrothermal approach using SiO_2_ spheres as self‐sacrificial templates.^[^ ^19^ ^]^ Then, air‐treated CSHSs (CSHSs‐air) and argon‐treated CSHSs (CSHSs‐Ar) were developed with the assistance of high‐temperature calcination under air or argon condition. As shown in Figure **S1** (Supporting Information), monodispersed SiO_2_ spheres held an average size of 420 nm with a uniform distribution of Si and O. Both scanning electron microscope (SEM) and transmission electron microscope (TEM) images indicated that CSHSs, CSHSs‐air, and CSHSs‐Ar held hollow urchin‐like morphology with radially aligned spikes on their surfaces (Figure 1B–D). By counting at least 100 particles, CSHSs, CSHSs‐air, CSHSs‐Ar were found to have similar average sizes of 440 nm (Figure S2, Supporting Information). It was worthy of note that the presence of small particles in CSHSs‐Ar could be ascribed to the formation of Cu_2_O during the argon‐assisted high‐temperature calcination, which were marked with white arrows in both SEM and TEM images. Energy‐dispersive spectroscopy (EDS) and corresponding elemental mapping images further suggested the uniform distribution of Cu, Si, and O in above samples (Figure 1B–D; Figure S3, Supporting Information). Elemental composition and chemical state of copper in CSHSs, CSHSs‐air, and CSHSs‐Ar were further evaluated using X‐ray photoelectron spectroscopy (XPS). As expected, the signals of Cu 2p, Si 2p, and O 1s were easily found in the XPS spectra of above three samples (Figure S4A,B, Supporting Information). Characterized by the high‐resolution XPS spectra, four fitting peaks at 932.9, 935.8, 952.7, and 955.6 eV could be attributed to the Cu^+^ 2p3/2, Cu^2+^ 2p3/2, Cu^+^ 2p1/2, and Cu^2+^ 2p1/2, respectively (Figure 1E).^[^ ^20^ ^]^ Meanwhile, other peaks were the satellite peaks (Sat.) of Cu^2+^. These results thus implied the presence of Cu^+^ in CSHSs‐Ar and the absence of Cu^+^ in CSHSs and CSHSs‐air. Moreover, two fitting peaks at 102.8 and 103.5 eV based on high‐resolution Si 2p spectra of the above samples could be assigned to silicate (Si 2p_3/2_) and SiO_2_ (Si 2p_1/2_), respectively. Compared with CSHSs and CSHSs‐air, the increase of Si 2p_3/2_ percentage and decrease of Si 2p_1/2_ percentage in CSHSs‐Ar further demonstrated the increase of silicate content after the argon treatment. In addition, ^29^Si NMR spectra revealed that all the prepared samples had a sharp signal at 111.8 ppm with high signal to noise (S/N) ratio, further suggesting the presence of 3D networks with four oxygen bridges in CSHSs, CSHSs‐air, and CSHSs‐Ar. (Figure S4C, Supporting Information). Quantitatively, the Cu^+^ in CSHSs‐Ar could be calculated as 58.89% (Table S1, Supporting Information). All these exciting results suggested the occurrence of electron transfer from Si atoms to Cu atoms and reduction reaction from Cu^2+^ to Cu^+^ in the typical synthesis of CSHSs‐Ar. X‐ray diffraction (XRD) patterns were then utilized to verify the crystal structure of the above samples. As expected, the XRD patterns of CSHSs and CSHSs‐air well matched the standard peaks of copper silicate (JCPDS No. 03–0219), indicating that the air treatment did not cause the change of crystal structure of CSHSs (Figure 1F). Different from CSHSs and CSHSs‐air, CSHSs‐Ar held other diffraction peaks at 29.58°, 36.44°, 42.33°, and 61.41°, which could be assigned to the (110), (111), (200), and (220) facets of Cu_2_O (JCPDS No. 78–2076).^[^ ^21^ ^]^ According to the high‐resolution TEM (HR‐TEM) image, measured interplanar spacings of 0.3, 0.24, 0.21, and 0.15 nm could be indexed to the crystal planes of (110), (111), (200), and (220) of Cu_2_O, which was highly consistent with both XRD pattern and selected area electron diffraction (SAED) result (Figure S5A, Supporting Information). Furthermore, the chemical state and coordination environment of Cu in CSHSs‐Ar were explored using X‐ray absorption spectroscopy (XAS) technique.^[^ ^17d,e^ ^]^ According to the observed Cu K‐edge X‐ray absorption near edge structure (XANES) spectra of CSHSs‐Ar and their references, the absorption edge of CSHSs‐Ar was between CuO and Cu_2_O, which suggested the valence state of Cu was higher than + 1 but lower than + 2 (Figure 1G). Meanwhile, the results of Fourier‐transformed (FT) k^3^‐weighted extended X‐ray absorption fine structure (EXAFS) spectra demonstrated that there was only an enhanced peak at ≈1.35 Å, which could be assigned to the Cu‐O bonds (Figure 1H). In addition, the Cu‐Cu bond at ≈2.2 Å was negligible, implying that nearly all the Cu atoms in CSHSs‐Ar were well coordinated by O atoms. Figure 1I provided the Cu K‐edge wavelet transform (WT) EXAFS results of CSHSs‐Ar and their references. As expected, a visual representation could effectively illustrate that the high‐intensity zone of CSHSs‐Ar occupied a wider range than Cu_2_O and CuO, which originated from the joint contribution of Cu‐O bonds. The above experimental results based on XAS technique closely correlated with our previous XPS analysis of CSHSs‐Ar. Evidenced by the Fourier transform infrared (FT‐IR) spectra, all these three samples had similar functional groups (Figure S5B, Supporting Information). In detail, the peaks around 810 and 1 040 cm^−1^ could be attributed to the symmetrical stretching vibration and asymmetric stretching vibration of Si‐O. Together, these results confirmed the successful synthesis of CSHSs‐Ar with well‐defined morphology and high amount of Cu^+^ component.

### 2.2 POD‐Like Catalytic Activity of CSHSs‐Ar

**Figure 2:**
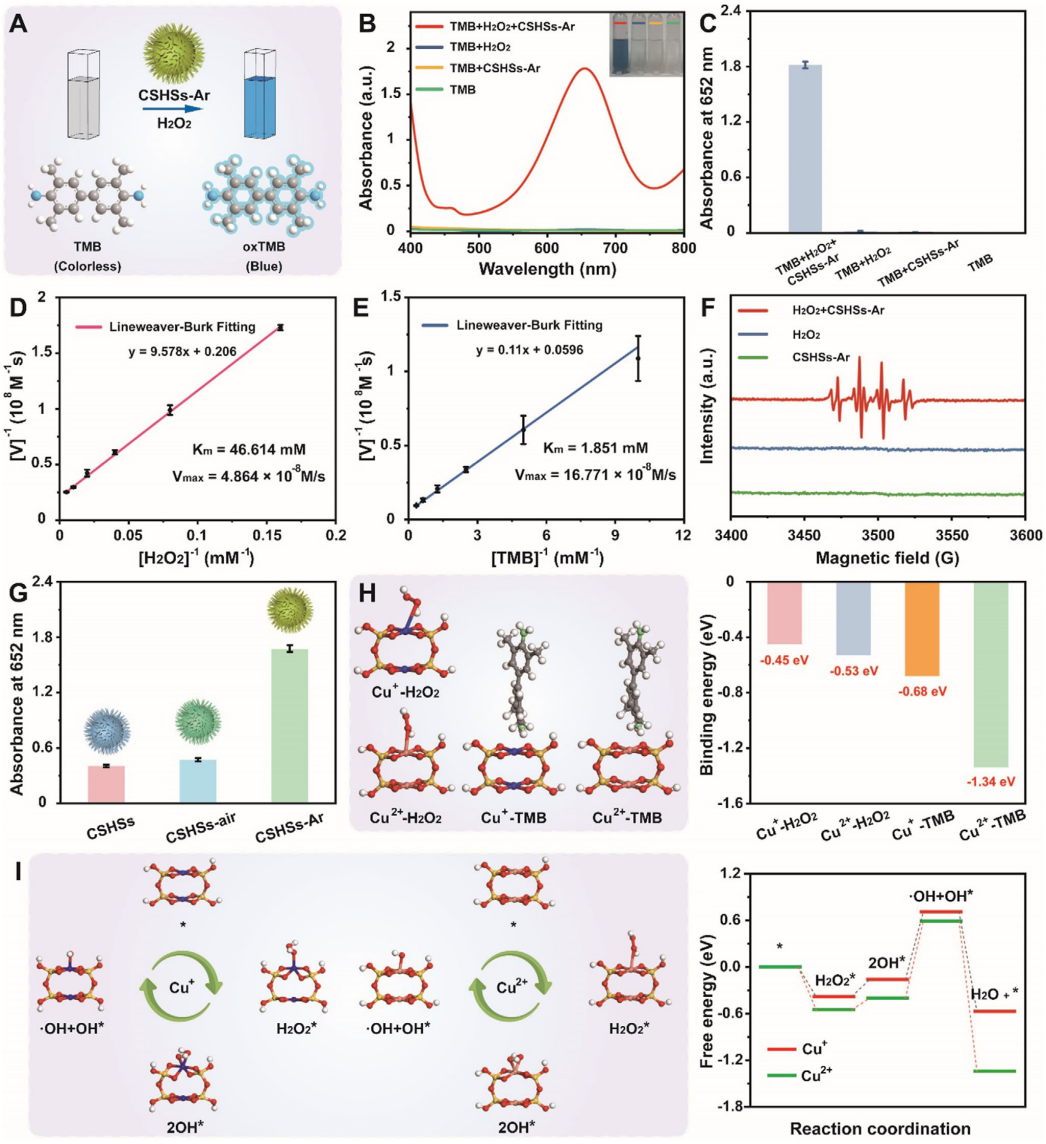
A) Schematic illustration of the POD‐like catalytic activity of CSHSs‐Ar. B) UV–vis absorbance spectra and C) quantitative information of the system catalyzed by CSHSs‐Ar. Inset of B: corresponding photos of different samples. In the typical experiment, the value of OD_652 nm_ of phosphate buffered saline (pH 4.0, 20 mm, 3 mL) containing TMB (1 mm), H_2_O_2_ (20 mm), and CSHSs‐Ar (100 µg mL⁻^1^) was recorded after the co‐incubation. Double‐reciprocal plots of POD‐like catalytic activity of CSHSs‐Ar at a fixed concentration of one substrate versus different concentration of the second substrate for D) H_2_O_2_ or E) TMB. F) ESR spectra for the detection of ·OH using DMPO as a capture reagent. G) Comparison of the POD‐like catalytic activity of CSHSs, CSHSs‐air, and CSHSs‐Ar. H) Geometry structure and corresponding binding energy of H_2_O_2_ and TMB adsorbed on copper with different valence states. I) Schematic illustration of the proposed catalytic mechanism of CSHSs‐Ar for the generation of ·OH and the free‐energy diagrams of catalytic pathways. Data in (C), (D), (E), and (G) were presented as mean ± SD (*n* = 3).

As illustrated in **Figure** **2**A, the POD‐like catalytic activity of CSHSs‐Ar was studied using typical TMB colorimetric method.^[^ ^22^ ^]^ UV–vis absorbance spectra and corresponding quantitative results showed that when CSHSs‐Ar were mixed with H_2_O_2_ and TMB, a typical dark blue color could be found with a clear absorbance at 652 nm (Figure 2B,C). However, without the addition of one of the components (H_2_O_2_ or CSHSs‐Ar), no visible blue color appeared. Above results indicated the highly intrinsic POD‐like catalytic activity of CSHSs‐Ar, which could efficiently catalyze H_2_O_2_ into ·OH and oxidize the colorless TMB into blue oxTMB. Since temperature and pH value seriously affected the POD‐like catalytic activity of any newly developed POD mimetics, the optimal reaction condition for our CSHSs‐Ar+H_2_O_2_ system was well studied. As shown in Figure S6 (Supporting Information), CSHSs‐Ar had the highest POD‐like catalytic activity at 50 °C (from 20 to 50 °C) and a pH value of 4.0 (from 2.0 to 9.0). Figure S7 (Supporting Information) implied that the POD‐like catalytic activity of CSHSs‐Ar also followed a concentration‐dependent manner whereas the absorbance at 652 nm was highly correlated with the levels of various components including CSHSs‐Ar, H_2_O_2_, and TMB. In order to assess the catalytic efficiency of CSHSs‐Ar, the maximum initial velocity (V_max_) and Michaelis‐Menten constant (K_m_) were calculated using the Lineweaver‐Burk equation at varied concentrations of H_2_O_2_ or TMB. In the case of H_2_O_2_ substrate, K_m_ and V_max_ were 46.614 mm and 4.864 × 10^−8^ m s⁻^1^, whereas for TMB substrate, K_m_ and V_max_ were 1.851 mm and 16.771 × 10^−8^ m s⁻^1^ (Figure 2D,E; Figure S8 and Table S2, Supporting Information). Notably, the K_m_ value of CSHSs‐Ar was much lower than that of classical Fe_3_O_4_ nanozyme with a K_m_ value of 154 mm, suggesting the stronger affinity between CSHSs‐Ar and H_2_O_2_.^[^ ^23^ ^]^ Also, CSHSs‐Ar held better affinity toward H_2_O_2_ or TMB than CSHSs previously studied by us. Subsequently, an electron spin resonance (ESR) spectrometer was utilized to explore the catalytic mechanism of our CSHSs‐Ar+H_2_O_2_ system. Captured by DMPO, typical ·OH signal could be easily found, indicating the generation of ·OH in our above‐mentioned system (Figure 2F). Furthermore, ABTS and OPD were utilized as another two chromogenic substrates to re‐confirm our hypothesis. As shown in Figure S9 (Supporting Information), both ABTS and OPD could be catalytically oxidated into color products with typical absorption peaks at 417 nm and 430 nm, respectively.

With the assistance of TMB as the typical substrate, the comparison of POD‐like catalytic activity of CSHSs, CSHSs‐air, and CSHSs‐Ar was further investigated. As shown in Figure 2G, CSHSs‐Ar held the highest absorbance value at 652 nm among all the samples, implying its best POD‐like catalytic activity. Quantitatively, the POD‐like catalytic activity of CSHSs‐Ar was 4.24 times that of CSHSs and 3.67 times that of CSHSs‐air, respectively. Meanwhile, CSHSs and CSHSs‐air held nearly the same absorbance values at 652 nm, indicating their similar POD‐like catalytic activity. The slightly higher POD‐like catalytic activity of CSHSs‐air over CSHSs could be ascribed to the weight loss during the synthesis of CSHSs‐air, which was confirmed by the thermogravimetric analysis (Figure S10, Supporting Information). Similar with some previous studies including ours, above prominent catalytic activity of CSHSs‐Ar over other two POD mimetics might originate from the abundant presence of Cu^+^ in CSHSs‐Ar, which not only held better catalytic performance over Cu^2+^, but also exhibited more advantages in promoting the activation of Cu^+^/Cu^2+^ redox cycling.^[^ ^24^ ^]^ To achieve a better understanding of above hypothesis, density functional theory (DFT) calculation was employed to explore the adsorption behavior of various chemical intermediates. Geometry structures of Cu^+^ and Cu^2+^ were illustrated in Figure S11 (Supporting Information) and CSHSs‐Ar centered on copper was well structured as models at first. Figure 2H showed that energy barrier associated with the adsorb of H_2_O_2_ onto Cu^2+^ was much lower than Cu^+^, which suggested that the catalytic activation of H_2_O_2_ on Cu^+^ was much easier. Meanwhile, the binding energy of Cu^2+^‐TMB was also lower than that of Cu^+^‐TMB, implying that the composite of Cu^2+^‐TMB was stabler and the decomposition of Cu^2+^‐TMB was more difficult compared with Cu^+^‐TMB, which re‐confirmed that Cu^+^ in CSHSs‐Ar might hold higher catalytic activity than Cu^2+^ while CSHSs‐Ar exhibited better POD‐like catalytic activity than CSHSs and CSHSs‐air. Figure 2I illustrated the catalytic mechanism of CSHSs‐Ar for the generation of ·OH and summarized the free‐energy diagrams of catalytic pathways. According to our design, the following steps might occur on the central sites of CSHSs‐Ar including Cu^+^ and Cu^2+^. First, H_2_O_2_ adsorbed onto the surface of CSHSs‐Ar, which resulted in the formation of *H_2_O_2_. Second, adsorbed H_2_O_2_ performed dissociation, leading to the creation of two *OH. Third, one *OH disengaged from the surface of CSHSs‐Ar as a ·OH. Finally, the remaining *OH became protonated toward the generation of *H_2_O, which could subsequently be released from the surface. Together, the above four steps well completed the catalytic cycle. It was worth noting that the overall rate of catalytic reaction was mainly determined by the formation of ·OH at central sites of CSHSs‐Ar, which required an energy of 0.87 eV for Cu^+^ and an energy of 0.99 eV for Cu^2+^, respectively. The above‐mentioned energy requirement demonstrated that the formation of disengaged ·OH from *OH adsorbed at Cu^+^ site was easier than that of Cu^2+^ site, further implying that Cu^+^ in CSHSs‐Ar exhibited better POD‐like catalytic activity than Cu^2+^. All these exciting results thus indicated that tuning the composition of POD‐like nanozymes could well endow them with efficient catalytic activity and specificity, which could be regarded as one of the high‐priority research directions of nanozymes in the future.

### 2.3 Colorimetric Detection of Typical VSCs and Thioalcohols

**Figure 3:**
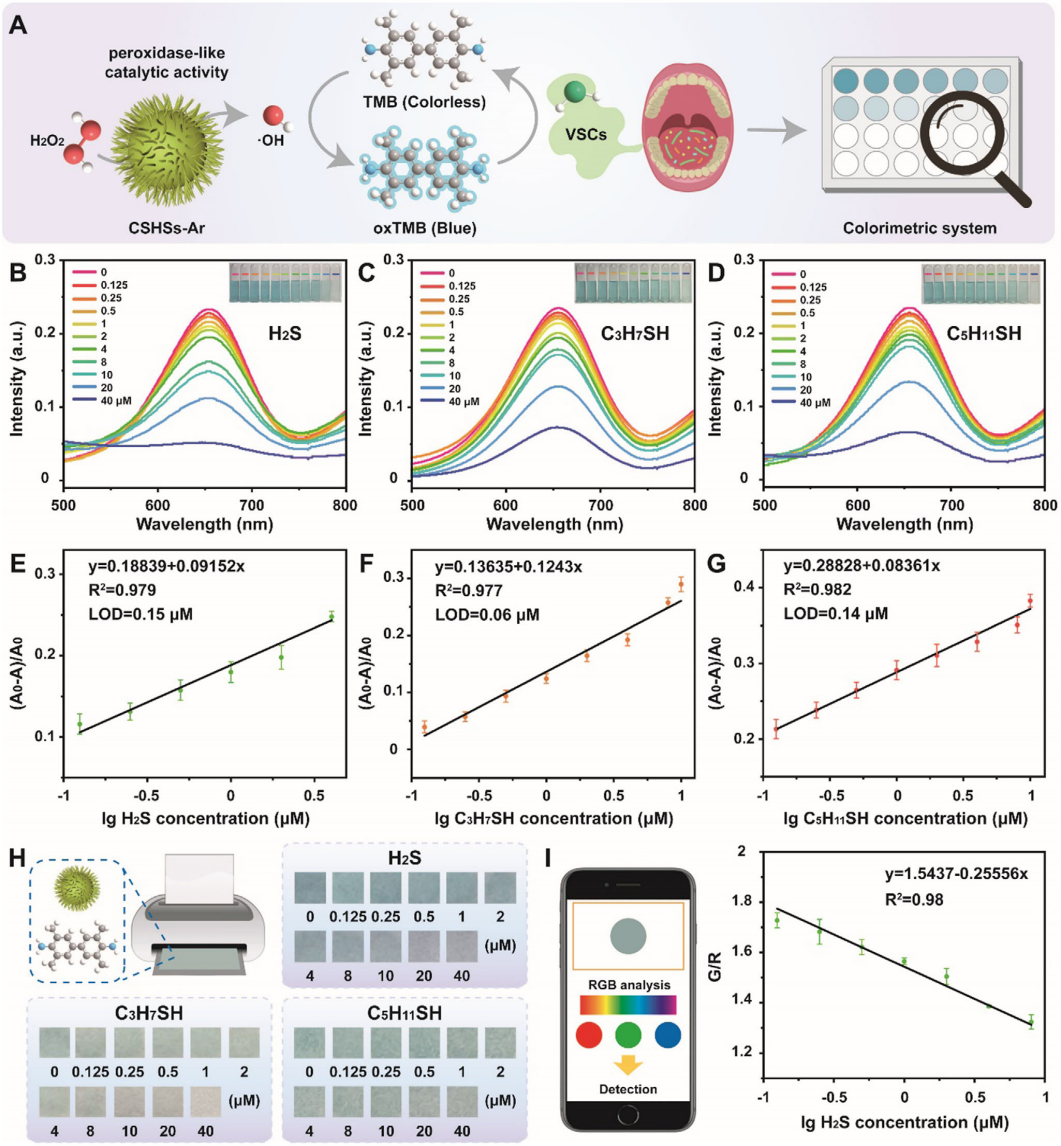
A) Schematic illustration for the detection of typical VSCs using CSHSs‐Ar nanozyme‐involved system. Concentration‐dependent UV–vis absorbance spectra of our detection system for B) H_2_S, C) C_3_H_7_SH, and D) C_5_H_11_SH. Inset of B–D: corresponding photos of the system treated with H_2_S, C_3_H_7_SH, and C_5_H_11_SH. In the typical experiment, the values of OD_652 nm_ of phosphate buffered saline (pH 4.0, 20 mm, 3 mL) containing TMB (1 mm), H_2_O_2_ (20 mm), CSHSs‐Ar (15 µg mL⁻^1^), and H_2_S/C_3_H_7_SH/C_5_H_11_SH were recorded after co‐incubation. Linear curves for E) H_2_S, F) C_3_H_7_SH, and G) C_5_H_11_SH with different concentration ranges. H) Schematic illustration for the preparation of colorimetric paper and corresponding discoloration photos of paper sensor in the detection of H_2_S, C_3_H_7_SH, and C_5_H_11_SH. I) Schematic illustration for the H_2_S detection using smartphone color recognizer and the plot of color change (green/red) versus concentration. Data in (E), (F), (G), and (I) were presented as mean ± SD (*n* = 3).

The detection of typical VSCs and thioalcohols using our well‐developed CSHSs‐Ar nanozyme‐involved system was illustrated in **Figure** **3**A. As well known, periodontitis‐related oral pathogens could produce VSCs including H_2_S during their metabolism process, which could be considered as the majority of halitosis‐forming gases.^[^ ^25^ ^]^ The detection results and corresponding quantitative information of colorimetric sensing of H_2_S and thioalcohols were shown in Figure 3B–G, Figure S12 and Table S3 (Supporting Information). With the addition of H_2_S, the value of absorbance at 652 nm decreased gradually with a color change from blue to colorless, indicating that oxTMB was reduced to the colorless TMB. We then investigated the linear relationship between the concentration of H_2_S and thioalcohols and the relative absorbance intensity (A_0_‐A)/A_0_, in which A represented the absorbance value measured at a certain concentration and A_0_ represented the absorbance value of the blank control). Figure 3E indicated a concentration response curve for H_2_S with an equation of y = 0.18839 + 0.09152x (R^2^ = 0.979) in a range of 0.125‐4 µm and a limit of detection (LOD) of 0.15 µm (3σ/slope). By replacing H_2_S with 1‐propanethiol (C_3_H_7_SH) and 1‐amyl mercaptan (C_5_H_11_SH), similar results could be easily found according to our experimental results. As expected, our detection system also held a good linear response for C_3_H_7_SH and C_5_H_11_SH. Figure 3F revealed a concentration response curve for C_3_H_7_SH with an equation of y = 0.13635 + 0.1243x (R^2^ = 0.977) in a range of 0.125–4 µm and a LOD of 0.06 µm. Meanwhile, the concentration response curve for C_5_H_11_SH was fitted as y = 0.28828 + 0.08361x (R^2^ = 0.982, 0.125–4 µm) with a LOD of 0.14 µm (Figure 3G). Moreover, the detection performance of our system toward H_2_S was comparable or even superior to those of previous Cu‐based nanozyme‐assisted sensors (Table S3, Supporting Information). All these results demonstrated the high outcomes of our system for the detection of typical VSCs and thioalcohols.

Considering the detection efficacy and accuracy, we further evaluated the selectivity of our colorimetric system for the detection of H_2_S and thioalcohols. The effects of amino acids without thiol group (Lys, Try, Gly, Thr, Glu), cations (Na^+^, K^+^, Ca^2+^, Mg^2+^, Zn^2+^), anions (F^−^, Cl^−^, SO_4_^2−^, PO_4_^3−^, NO_3_^−^), and other biomolecules (urea and glucose) on the selectivity of our system were well explored. Figure S13 (Supporting Information) indicated that our colorimetric system could selectively detect H_2_S and thioalcohols with admirable anti‐interference ability.

After understanding the high selectivity of our system, a smartphone platform with the assistance of colorimetric paper was developed for the visual detection of H_2_S and thioalcohols. The test strips were prepared by printing colorimetric solution on filter paper at first. When paper strips were immersed into acidic phosphate buffered saline containing thioalcohols and H_2_O_2_, their color changed quickly and tended to be stable within 10 min. As shown in Figure 3H, the color changes of paper strips treated with different concentrations of H_2_S and thioalcohols could be visually detected. Then, digitized images of the above paper strips were captured using a smartphone and corresponding RGB (Red/Green/Blue) data were analyzed upon a color recognition software.^[^ ^26^ ^]^ Figure 3I indicated that the linear relationship (R^2^ = 0.98, 0.125–8 µm) was well fitted and the LOD of H_2_S could be calculated as 0.44 µm. Thus, our strategy of integrating colorimetric paper with smartphone could well complete the visual and quantitative detection of typical VSCs including H_2_S.

As typical biothiols, L‐cysteine (Cys) and glutathione (GSH) played essential roles during the normal physiological process.^[^ ^27^ ^]^ The possibility of CSHSs‐Ar nanozyme‐involved system in the detection of Cys and GSH was further assessed. As shown in Figure S14 (Supporting Information), there was a good linear relationship (R^2^ = 0.978 or 0.993) between the absorbance value of detection system and the concentration of Cys (0.125–10 µm) or GSH (0.125–80 µm) with a low LOD (0.15 or 0.07 µm), respectively. Meanwhile, we summarized some Cu‐based nanozyme‐assisted sensors focused on the detection of Cys and GSH in recent years (Tables S4 and S5, Supporting Information). It could be easily found that our well‐developed nanoplatform held more satisfactory detection outcomes. All these results indicated the feasibility of our system in the detection of other thiol‐contained reagents including classical biothiols.

To better understand the effect of VSCs against the production of ·OH during the detection, FT‐IR measurement was utilized to provide additional evidence for the catalytic mechanism. Figure S15A (Supporting Information) provided the detailed experimental protocol and the corresponding FT‐IR spectra of samples after different treatments. Considering the volatility of thiols and the convenience of detection, C_3_H_7_SH with high concentration was selected and utilized as the typical VSC in our current design. Figure S15B (Supporting Information) revealed that there was no significant difference in FT‐IR spectra between CSHSs‐Ar and CSHSs‐Ar+H_2_O_2_. After the addition of C_3_H_7_SH, several new characteristic peaks around 3 000 cm^−1^ were easily found, which could be ascribed to the C‐H stretching vibrations of ‐CH_2_‐ and ‐CH_3_. Because of the strong interaction between Cu sites and thiol groups, the residual C_3_H_7_SH could be well adsorbed on the surface of CSHSs‐Ar after reaction, further leading to the above‐mentioned characteristic peaks in FT‐IR spectra. Together, VSCs could well react with the generated ·OH during the colorimetric detection, which was suggested as the main mechanism of our current design.

### 2.4 Visual Logic Gates

**Figure 4:**
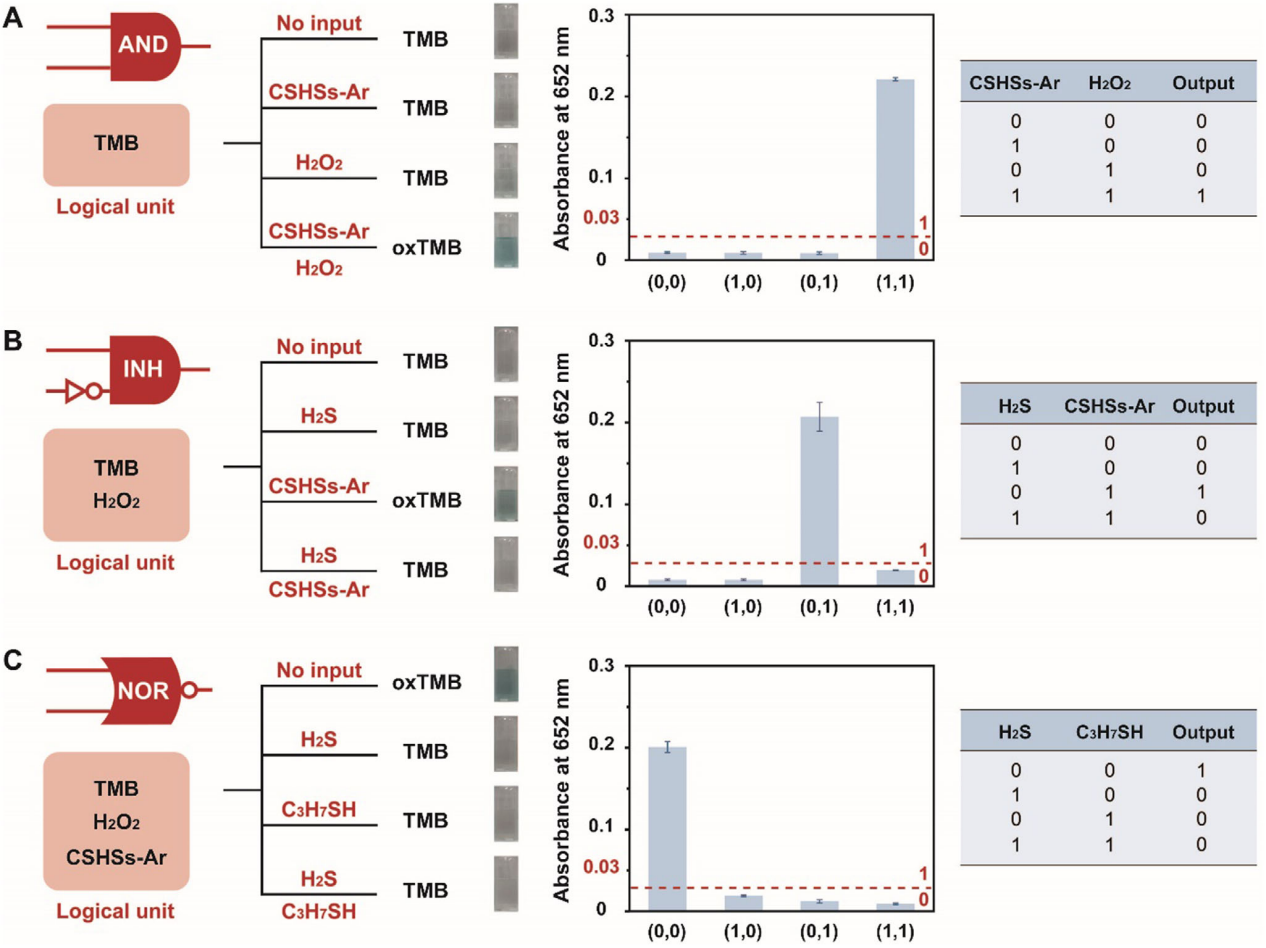
Logic scheme, photos of the samples treated with different inputs, UV–vis absorbance intensities at 652 nm, and truth table for A) AND gate, B) INH gate, and C) NOR gate. Data were presented as mean ± SD (*n* = 3).

In addition to colorimetric detection of typical VSCs, thioalcohols, and biothiols, our system could be employed for the design of visual logic operations. In general, bio‐related visual logic operations could be regarded as essential components in the fields of disease diagnosis, tumor therapy, and drug delivery, which exhibited high potentials in both medical application and clinical translation.^[^ ^28^ ^]^ For all the logic gates, the absorbance of system at 652 nm was regarded as the output with a threshold value of 0.03. In general, an AND gate was represented by the condition in which the output of gate was 1 only if both inputs were 1. Using TMB as the logic unit, CSHSs‐Ar and H_2_O_2_ served as the two inputs. In general, the absence of either input was defined as 0 while the presence of either input was defined as 1. **Figure** **4**A revealed that only in the presence of both CSHSs‐Ar and H_2_O_2_ (1, 1), a significant increase in OD_652 nm_ could be detected (output = 1), suggesting that the system behaved as an AND logic gate. Meanwhile, other possible inputs of (0, 0), (1, 0), and (0, 1) only resulted in a weaker or none absorption at 652 nm without obvious color change. Using logic unit consisting of TMB and H_2_O_2_, an INH logic gate was also developed while CSHSs‐Ar and H_2_S acted as inputs. The introduction of H_2_S could highly inhibit the interaction between TMB and H_2_O_2_ (Figure 4B). There was an output signal of 1 only if the CSHSs‐Ar was involved into the logic unit (0, 1). Using TMB, H_2_O_2_ and CSHSs‐Ar as the logic unit, an NOR logic gate was further created. Figure 4C indicated that H_2_S, C_3_H_7_SH, and their mixture could efficiently neutralize H_2_O_2_ and decrease the color change of logic unit. On this basis, the output signal of 1 corresponding to blue color could be achieved only when both inputs were absent. These results implied that our system could also acquire great results in the development of visual logic gates owing to its great efficiency.

### 2.5 Visual Detection of Periodontitis

**Figure 5:**
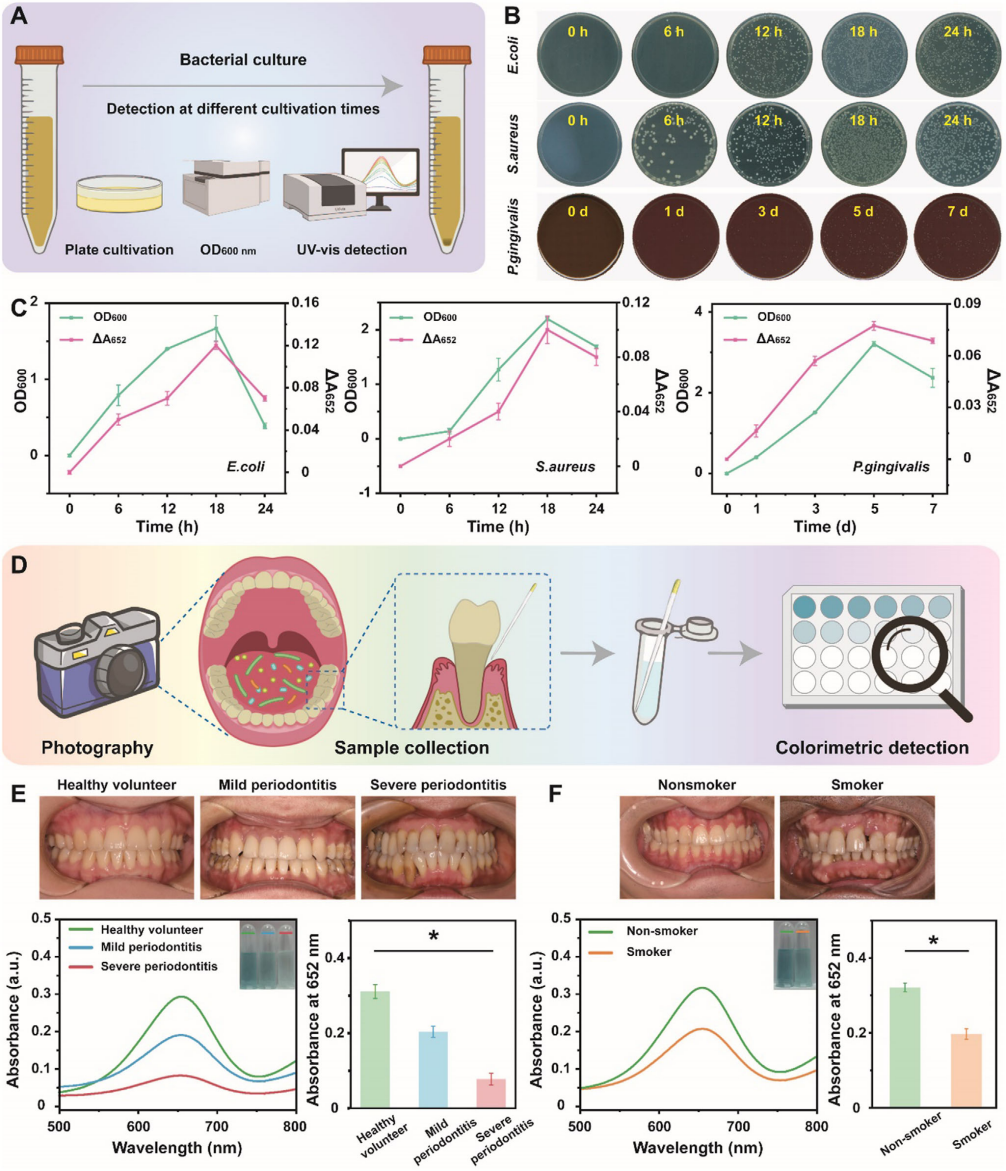
A) Schematic illustration for the detection approaches of bacterial proliferation. B) Co‐incubation period‐dependent photos of CFU plating of various bacteria and C) the relationship between the OD_600 nm_ of bacterial suspensions and the ΔA_652_ of culture medium according to our detection strategy. D) Schematic illustration for the GCF collection from the volunteers with different periodontal status and corresponding colorimetric detection. Photos and colorimetric detection results for the volunteers with different degrees of E) periodontitis and F) smoker/non‐smoker. Data in (C), (E), and (F) were presented as mean ± SD (*n* = 3). Statistical significance in (E) was calculated using one‐way analysis of variance (ANOVA). *p < 0.05. Statistical significance in (F) was calculated using unpaired *t*‐test. *p < 0.05.

The high sensitivity and great selectivity of CSHSs‐Ar nanozyme‐involved system suggested its performance in the visual detection of VSCs produced by oral pathogens. Experiments involving bacterial culture and relative detection of growth curves were utilized to confirm our hypothesis (**Figure** **5**A).^[^ ^29^ ^]^ In detail, ager plates showed the growth of bacteria at different incubation time points while OD_600 nm_ was tested for each bacterial medium. Meanwhile, UV–vis adsorption spectra were utilized for real‐time monitoring the generation of VSCs during the whole process of bacterial culture. As expected, the number of above bacteria cultured on the agar plates increased along with the time passing (Figure 5B; Figures S16 and S17, Supporting Information). Colorimetric assay suggested a gradual increase in the production of VSCs within the first 18 h for both *E. coli* and *S. aureus*, as well as a decline after 18 h due to the competitive inhibition on bacteria, which held a similar result with the OD_600 nm_ values of bacterial liquid culture (Figure 5C; Figure S18, Supporting Information). As a crucial oral pathogen responsible for periodontitis, *P. gingivalis* could produce VSCs during their metabolism. Accordingly, the performance of our system in the detection of VSCs induced by *P. gingivalis* was further evaluated as the approach described above. Similar with the trend of bacterial growth curve, *P. gingivalis*‐generated VSCs increased in the first 5 days and then decreased. The above results suggested the reliability of CSHSs‐Ar nanozyme‐involved system for monitoring the growth and metabolism of oral pathogens via the detection of typical VSCs.

To confirm the feasibility of our approach in testing clinical samples, gingival crevicular fluid (GCF) containing VSCs was collected before the visual detection.^[^ ^30^ ^]^ Figure 5D illustrated the strategy based on CSHSs‐Ar nanozyme‐involved system for the diagnosis of periodontitis and other periodontal status. After the addition of GCF samples from healthy volunteers or patients with periodontitis into our detection system, the absorbance values at 652 nm of different samples were carefully recorded. Due to the abundance of bacteria in the oral cavity of patients with periodontitis and the strong reduction ability of VSCs, samples containing the GCF from healthy volunteers held the highest absorbance at 652 nm while samples containing the GCF from patients with severe periodontitis had the lowest ones (Figure 5E). Since smoking could influence oral condition and contribute to halitosis and periodontal disease, GCF samples from smokers and non‐smokers were then collected to verify the performance of our detection system.^[^ ^31^ ^]^ Figure 5F revealed that the concentration of VSCs in the GCF from smokers was much lower than those from non‐smokers. In addition, GCF samples from volunteers before and after toothbrushing were also tested.^[^ ^32^ ^]^ Figure S19 (Supporting Information) indicated that efficient toothbrushing could extremely decrease the amount of VSCs in oral cavity. All these results not only suggested the desirable sensitivity of CSHSs‐Ar nanozyme‐involved system toward GCF samples, but also indicated the reliability of our detection system for the monitoring of different periodontal status.

### 2.6 Biosafety Evaluation of CSHSs‐Ar

**Figure 6:**
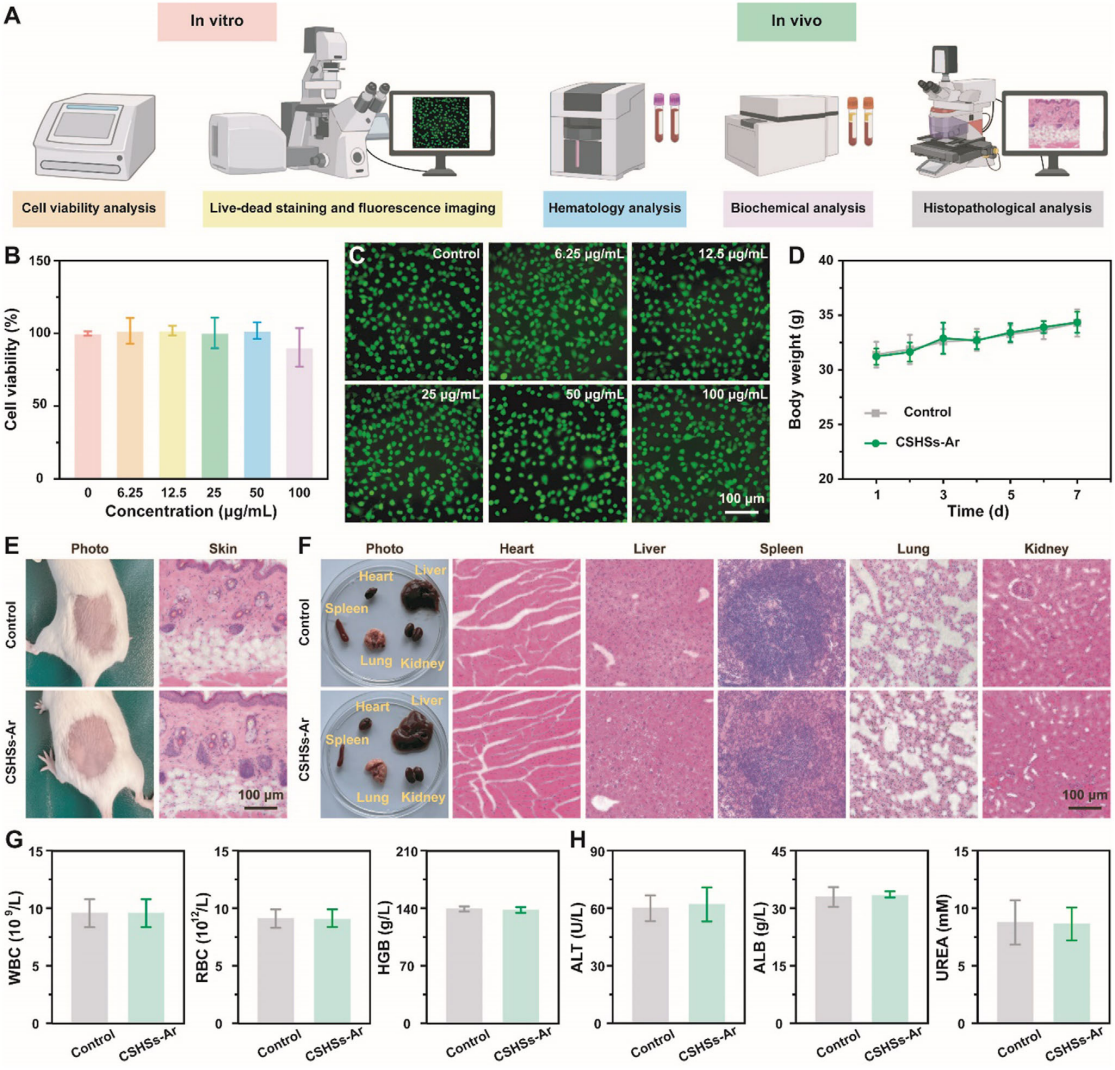
A) Schematic illustration for the biosafety evaluation of CSHSs‐Ar. B) Viabilities and C) live/dead staining images of L929 cells after being co‐incubated with CSHSs‐Ar. D) Body weight changes, E) photos and H&E‐stained images of skin, F) photos and H&E‐stained images of main organs, G) hematological index, and H) blood biochemical index of mice after the skin exposure of CSHSs‐Ar. Data in (B), (G), and (H) were presented as mean ± SD (*n* = 3). Data in (D) were presented as mean ± SD (*n* = 5).

Considering the possible risk of reagent exposure during the colorimetric detection, we further evaluated the biosafety of CSHSs‐Ar. Detailed experimental design was illustrated in **Figure** **6**A. According to the results of cell viability analysis and live/dead staining images, CSHSs‐Ar showed no obvious growth inhibition on L929 cells even at a high concentration of 100 µg mL⁻^1^ (Figure 6B,C). Meanwhile, the expression levels of interleukin‐6 (IL‐6) in the cell culture medium were measured, which could indicate the presence of pathological conditions including infection, inflammation, and autoimmune diseases. As shown in Figure S20 (Supporting Information), low concentration of H_2_O_2_ exhibited extremely low cytotoxicity, which could be utilized as a positive control in the following experiment. Quantitatively, the release amounts and relative percentages of IL‐6 from L929 cells after the co‐incubation with different concentrations of CSHSs‐Ar were much lower than that of H_2_O_2_, further suggesting that CSHSs‐Ar could not induce serious cellular inflammatory response. Furthermore, we explored the in vivo toxicity of CSHSs‐Ar after the sufficient skin exposure. During the one‐week experimental period, mice in the two groups gained body weight gradually and followed a same growth trend (Figure 6D). Photos and hematoxylin and eosin (H&E)‐stained images of skins and main sensitive organs demonstrated that there were no significant differences between the two groups, implying that nearly no organ damage and skin inflammation response occurred after the skin exposure of CSHSs‐Ar (Figure 6E,F). Besides, results of hematology and blood biochemistry analysis suggested that the treatment of CSHSs‐Ar had no impact on the hematological index and blood biochemical index (Figure 6G,H). Together, all these findings indicated the overall biosafety of CSHSs‐Ar both in vitro and in vivo.

## 3 Conclusion

In summary, we rationally designed and developed a novel copper silicate nanozyme with enhanced POD‐like catalytic activity using a facile in situ valence‐engineered strategy. Results based on both experimental and theoretical investigations demonstrated that these CSHSs‐Ar nanozymes were more beneficial toward the activation of H_2_O_2_, promising their high POD‐like catalytic activity. Coupling with the negligible interference in detection, CSHSs‐Ar nanozyme‐involved system could serve as an efficient colorimetric sensor for the detection of VSCs and prediction for periodontitis. As expected, the current system with high sensitivity and selectivity held a wide detection range of various VSCs and low LODs around 0.1 µm. To meet the need for cost‐effective point‐of‐care testing, the visible detection of H_2_S was well achieved with the help of smartphone photography. Furthermore, our well‐developed system was successfully utilized to detect the trace VSCs in GCF samples for the accurate diagnosis of periodontitis with different degrees. In addition to the above‐mentioned application of CSHSs‐Ar nanozymes with superior POD‐like catalytic activity, we also established a series of visual molecular logic gates including AND, INH, and NOR as a proof of concept. Last but not least, these CSHSs exhibited high biosafety and low systemic toxicity both in vitro and in vivo. Taking together, our findings not only opened a new way for the development of nanozymes with high POD‐like catalytic activity using valence engineering, but also provided an innovative direction for the early diagnosis of clinical oral diseases.

## Conflict of Interest

The authors declare no conflict of interest.

## Supporting information



Supporting Information

## Data Availability

The data that support the findings of this study are available from the corresponding author upon reasonable request.
