# Supplementary material for: In Situ Valence Engineering of Copper Silicate Nanozymes with Enhanced Peroxidase‐Like Catalytic Activity for Oral Disease Detection
Source: Adv Sci (Weinh). 2025 Jun 5;12(33):e03237. doi: 10.1002/advs.202503237 (PMC12412579; doi:10.1002/advs.202503237)
Supplement: Supplementary file 1 — Supporting Information [file ADVS-12-e03237-s001.pdf]

ADVANCED SCIENCE
Open Access

Supporting Information

for *Adv. Sci.*, DOI 10.1002/advs.202503237

In Situ Valence Engineering of Copper Silicate Nanozymes with Enhanced Peroxidase-Like Catalytic Activity for Oral Disease Detection

Xiaocan Liu, Zhen Ding, Chengjing Xu, Jinming Zhang, Yufu Liu, Tianyan Chen, Shuang Dai, Xingfu Bao, Min Hu* and Zhen Liu**

Supporting Information for

In Situ Valence Engineering of Copper Silicate Nanozymes with Enhanced Peroxidase-Like Catalytic Activity for Oral Disease Detection

Xiaocan Liu, Zhen Ding, Chengjing Xu, Jinming Zhang, Yufu Liu, Tianyan Chen, Shuang Dai, Xingfu Bao, Min Hu,* and Zhen Liu**

I. Experimental Section

Chemicals. Tetraethyl orthosilicate (TEOS), glutathione (GSH), L-cysteine (Cys), L-lysine (Lys), DL-tryptophan (Try), Calcein AM, and propidium iodide (PI) were obtained from Sigma-Aldrich (Shanghai, China). $Cu(NO_3)_2{\cdot}3H_2O$, Cu_2O, CuO, Cu foil, $NH_3{\cdot}H_2O$ (25-28%), HCl, $Na_2S{\cdot}9H_2O$, urea, glucose, 1-propanethiol (C_3H_7SH), 1-amyl mercaptan ($C_5H_{11}SH$), 5,5-dimethyl-1-pyrroline N-oxide (DMPO), 2,2′-azino-bis (3-ethylbenzthiazoline-6-sulfonic acid) diammonium salt (ABTS), 3,3′,5,5′-tetramethylbenzidine (TMB), and o-phenylenediamine (OPD) were purchased from Aladdin Reagent (Shanghai, China). NaCl, NaF, Na_2SO_4, Na_3PO_4, $MgCl_2$, KCl, $CaCl_2$, $Zn(NO_3)_2$, H_2O_2, glycine (Gly), L-glutamic acid (Glu), and L-threonine (Thr) were purchased from Sinopharm Chemical Reagent (Shanghai, China). Dulbecco's modified Eagle's medium (DMEM) and fetal bovine serum (FBS) were obtained from ACRO Biosystems (Beijing, China). Cell Counting Kit-8 (CCK-8) was purchased from NCM Biotech (Suzhou, China). Mouse interleukin-6 (IL-6) enzyme-linked immunosorbent assay (ELISA) kit was purchased from Elabscience Biotech (Wuhan, China). All chemical reagents were of analytical grade and utilized without further purification. Deionized water through all experiments was obtained using a Milli-Q water system.

Instruments. The structure and morphology of various samples were characterized using a scanning electron microscope (SEM, Apreo 2C) and a high-resolution transmission electron microscope (TEM, Talos F200S). The chemical composition and crystal structure were tested on an X-ray photoelectron spectrometer (XPS, ESCALAB 250Xi) and an X-ray diffractometer (XRD, Rigaku Ultima IV), respectively. A Fourier transform infrared (FT-IR) spectrometer (NICOLET iS50) was utilized to record the FT-IR spectra of various samples. An electron spin resonance (ESR) spectrometer (Bruker E-500 ESR) was utilized to detect the generation of radicals in our system. Solid state ^{29}Si nuclear magnetic resonance (NMR) experiments were performed on a Bruker Avance III 400 MHz spectrometer. The X-ray absorption fine structure (XAFS) spectra were performed on a Table XAFS-500A instrument (Specreation Instruments).

Ultraviolet-visible (UV-vis) absorption spectra were recorded on a UV-vis spectrometer (Shimadzu UV-1800).

Synthesis of CSHSs, CSHSs-air, and CSHSs-Ar. Copper silicate hollow spheres (CSHSs) were synthesized using a facile two-step approach. Firstly, silica (SiO_2) spheres were prepared using a classical Stöber method. Then, CSHSs were developed *via* a hydrothermal method using SiO_2 spheres as self-sacrificial templates. Typically, TEOS (5 mL), $NH_3 \cdot H_2O$ (10 mL), ethanol (65 mL), and deionized water (25 mL) were mixed under stirring to produce SiO_2 spheres. For the synthesis of CSHSs, SiO_2 spheres (100 mg), $Cu(NO_3)_2 \cdot 3H_2O$ (0.75 mmol), and $NH_3 \cdot H_2O$ (1 mL) were dispersed in deionized water (50 mL), transferred to a Teflon-lined stainless-steel autoclave, and heated at 140 °C overnight. After cooling to room temperature, the products were rinsed with deionized water and ethanol in sequence, as well as freeze-dried in vacuum. In order to prepare air-treated CSHSs (CSHSs-air) and argon-treated CSHSs (CSHSs-Ar), the above CSHSs were treated with air or argon (Ar) at 800 °C overnight, respectively. The resulting products were stored in vacuum for further use.

Peroxidase (POD)-like catalytic activity of CSHSs-Ar. The POD-like catalytic activity of CSHSs-Ar was determined using TMB as the typical substrate in the presence of H_2O_2 at first. Then, the absorbance values at 652 nm ($OD_{652\ nm}$) of phosphate buffered saline (pH 4.0, 20 mM, 3 mL) containing TMB (1 mM), H_2O_2 (20 mM), and CSHSs-Ar (100 μg/mL) were recorded after the co-incubation at room temperature. Meanwhile, the pH/temperature-dependent POD-like catalytic activity of CSHSs-Ar was explored by changing the experimental pH values and temperatures. Moreover, the POD-like catalytic activity of CSHSs-Ar was re-confirmed using ABTS and OPD as the another two substrates with respective absorbance values at 417 nm ($OD_{417\ nm}$) and 430 nm ($OD_{430\ nm}$). Last but not least, the POD-like catalytic activities of CSHSs and CSHSs-air were investigated under a similar experimental condition.

Steady-state kinetic analysis. The steady-state kinetics were conducted at room temperature in phosphate buffered saline containing CSHSs-Ar, TMB, and H_2O_2. By using H_2O_2 as substrate,

$OD_{652\,nm}$ of phosphate buffered saline (pH 4.0, 20 mM, 3 mL) containing TMB (1 mM), CSHSs-Ar (100 μg/mL), and different concentrations of H_2O_2 were recorded with time. By using TMB as substrate, $OD_{652\,nm}$ of phosphate buffered saline (pH 4.0, 20 mM, 3 mL) containing H_2O_2 (20 mM), CSHSs-Ar (100 μg/mL), and different concentrations of TMB were recorded with time. Double reciprocal Michaelis-Menten curves were plotted and fitted to the following Lineweaver-Burk equations, in which V was the initial reaction velocity, V_{max} was the maximum reaction rate, [S] was the substrate concentration, and K_m was the Michaelis constant.

$$V = V_{max}\,\frac{[S]}{K_m + [S]}$$

ESR measurement. To determine the presence of reactive oxygen species in our system, ESR spectra were measured using DMPO as a typical capture reagent for the generated hydroxyl radical (·OH).

Density functional theory (DFT) calculation. DFT calculation was employed to explore the potential mechanism responsible for the catalytic reaction. All geometry optimizations were employed using DMol3 program from the DFT calculation.[1] The DFT Semi-core Pseudopots as type of treatment of the core electrons were treated in the calculation for Cu, whereas H, Si and O were performed as in the all-electron model. The double numerical plus polarization as the basis set was utilized, and the generalized gradient approximation with a Perdew-Burke-Ernzerh of method as the exchange-correlation functional was achieved.[2] To avoid the interaction between two neighboring images, the vacuum space along z direction was set to be 15 Å. The following convergence standard of total energy was 10^{-5} Ha, the force was 0.002 Ha/Å, and the maximum displacement tolerance was 0.005 Å. The adsorption energies were calculated according to the following formula, in which $E_{adsorbate/sub}$, $E_{adsorbate}$, and E_{sub} represented the total energy of substrate with adsorbed species, the adsorbate species, and the clean substrate, respectively.

$$E_{ads} = E_{adsorbate/sub} - E_{adsorbate} - E_{sub}$$

Colorimetric detection of typical VSCs, thioalcohols, and biothiols. Typically, phosphate buffered saline (pH 4.0, 200 mM, 300 μL), H_2O_2 (200 mM, 300 μL), TMB (10 mM, 300 μL), and CSHSs-Ar (1 mg/mL, 45 μL) were mixed with typical VSCs, thioalcohols, or biothiols at first. Subsequently, deionized water was utilized to dilute the above mixture to 3 mL. After the co-incubation at room temperature, the values of $OD_{652\ nm}$ of above samples were recorded.

Preparation of paper sensor and their usage in the detection of VSCs. In order to develop a paper sensor, solution containing TMB and CSHSs-Ar was utilized as ink and printed onto filter paper. Subsequently, the created colorimetric papers were cut into paper strips for further use. Typically, paper strips were immersed into acidic phosphate buffered saline containing H_2O_2 and VSCs, dried at room temperature, and photographed with a smartphone under a stable light source. Meanwhile, initial colorimetric paper and filter paper without any treatment served as the controls. With the help of the color recognition software in smartphone, the color change of paper sensor and corresponding RGB data were analysed. Furthermore, our detection was extended to the detection of Cys and GSH.

The selectivity of CSHSs-Ar. To assess the selectivity of our current design, the values of $OD_{652\ nm}$ were determined in the presence of different interferents including cations (Na^+, K^+, Ca^{2+}, Mg^{2+}, and Zn^{2+}), anions (F^-, Cl^-, SO_4^{2-}, PO_4^{3-}, and NO_3^-), amino acids (Lys, Try, Gly, Thr, and Glu), and other biomolecules (urea and glucose). The concentrations of VSCs, interference biomolecules, and interference ions in the typical experiments were 40, 100, and 400 μM.

Logic gates. All logic gates and corresponding experiments were well designed and performed at room temperature by using the signals of $OD_{652\ nm}$ as the outputs.

AND logic gate. TMB (10 mM, 300 μL) was added into acidic phosphate buffered saline (200 mM, 300 μL) at first. Then, the chromogenic reaction was initiated by four inputs. (0, 0): H_2O (2.4 mL); (1, 0): CSHSs-Ar (1 mg/mL, 40 μL) and H_2O (2.36 mL); (0, 1): H_2O_2 (200 mM, 300 μL) and H_2O (2.1 mL); (1, 1): CSHSs-Ar (1 mg/mL, 40 μL), H_2O_2 (200 mM, 300 μL), and H_2O (2.06 mL). The values of $OD_{652\ nm}$ were carefully recorded.

INH logic gate. TMB (10 mM, 300 μL) and H_2O_2 (200 mM, 300 μL) were added into acidic phosphate buffered saline (200 mM, 300 μL) at first. Then, the chromogenic reaction was initiated by four inputs. (0, 0): H_2O (2.1 mL); (1, 0): H_2S (10 mM, 100 μL) and H_2O (2 mL); (0, 1): CSHSs-Ar (1 mg/mL, 40 μL) and H_2O (2.06 mL); (1, 1): H_2S (10 mM, 100 μL), CSHSs-Ar (1 mg/mL, 40 μL), and H_2O (1.96 mL). The values of $OD_{652\ nm}$ were carefully recorded.

NOR logic gate. TMB (10 mM, 300 μL), H_2O_2 (200 mM, 300 μL), and CSHSs-Ar (1 mg/mL, 40 μL) were added into acidic phosphate buffered saline (200 mM, 300 μL) at first. Then, the chromogenic reaction was initiated by four inputs. (0, 0): H_2O (2.06 mL); (1, 0): H_2S (10 mM, 100 μL) and H_2O (1.96 mL); (0, 1): C_3H_7SH (10 mM, 100 μL) and H_2O (1.96 mL); (1, 1): H_2S (10 mM, 100 μL), C_3H_7SH (10 mM, 100 μL), and H_2O (1.86 mL). The values of $OD_{652\ nm}$ were carefully recorded.

Monitoring of the growth and proliferation of bacteria. *Staphylococcus aureus* (*S. aureus*, ATCC 29213) and *Escherichia coli* (*E. coli*, ATCC 25922) were cultured in Brain Heart Infusion (BHI) medium for 24 h while *Porphyromonas gingivalis* (*P. gingivalis*, ATCC 33277) were cultured in BHI medium containing Vitamin K and Hemin for 7 d. Every 6 h (for *S. aureus* or *E. coli*) or 1 d (for *P. gingivalis*), the culture medium was carefully taken for the following experiments including the measurement of $OD_{600\ nm}$, plate cultivation, as well as the detection of dissolved VSCs. For the detection of VSCs, acidic phosphate buffered saline (200 mM, 300 μL), H_2O_2 (200 mM, 300 μL), TMB (10 mM, 300 μL), and CSHSs-Ar (1 mg/mL, 40 μL) were mixed with the collected culture mediums at first. Then, deionized water was utilized to dilute the above mixture to 3 mL. After the co-incubation at room temperature, the values of $OD_{652\ nm}$ of above samples were carefully recorded. According to our detection system, ΔA_{652} was defined as the difference between the initial absorbance value of culture medium and the value of culture medium at each expected time-point.

Detection of clinical samples. With the approval of the Ethics Committee of the School and Hospital of Stomatology of Jilin University (SJDKQ2024004), gingival crevicular fluid (GCF)

samples were achieved after obtaining the informed consent of the subjects. Oral photos from the volunteers with different levels of periodontal status were taken, GCF samples were collected and diluted, as well as colorimetric detection was carried out using our detection strategy. For the detection of real samples, phosphate buffered saline (pH 4.0, 200 mM, 300 μL), H_2O_2 (200 mM, 300 μL), TMB (10 mM, 300 μL), and CSHSs-Ar (1 mg/mL, 60 μL) were mixed with the collected GCF samples from different volunteers at first. Then, deionized water was utilized to dilute the above mixture to 3 mL. After the co-incubation at room temperature, the values of $OD_{652\ nm}$ of above samples were carefully recorded.

Cell culture. L929 fibroblasts (L929 cells) were purchased from Chinese Academy of Medical Sciences and cultured in DMEM containing 10% FBS in a humidified incubator at 37 °C with 5% CO_2.

Cytotoxicity. CCK-8 assay was utilized to quantify the cytotoxicity of CSHSs-Ar. Briefly, L929 cells were seeded in a 96-well plate with a density of 1×10^4 cells per well. After the overnight culture, medium was replaced with fresh DMEM containing 10% FBS and different concentrations of CSHSs-Ar. Following a 12 h incubation, cell viability was evaluated using CCK-8 assay and calculated as the following formula.

$$Cell\ viability\ (\%\ of\ control) = \frac{OD_{sample} - OD_{blank}}{OD_{control} - OD_{blank}} \times 100$$

Visible cytotoxicity. Live-dead staining was utilized for the directed observation of cellular viability of L929 cells in the presence of CSHSs-Ar. L929 cells were seeded in a 6-well plate with a density of 1×10^6 cells per well at first. After the overnight culture, medium was replaced with fresh DMEM containing 10% FBS and different concentrations of CSHSs-Ar. Calcein AM and PI were utilized to treat L929 cells after the incubation, and images were captured with the help of a fluorescence microscope.

Detection of IL-6 in culture medium. L929 cells were seeded in a 96-well plate with a density of 1×10^4 cells per well. After the overnight culture, cells treated with H_2O_2 with a concentration

of 0.1 mM were considered as the positive control while cells without any treatments were defined as the negative control. Meanwhile, different concentrations of CSHSs-Ar were involved in the above experiment. Then, an ELISA kit was utilized to quantify the amounts of IL-6 in the cell culture medium.

Animals. Institute of Cancer Research (ICR) mice (female, 6-week old) were purchased from Jilin University Laboratory Animal Center (Changchun, China). All animal experimental procedures were approved by the Institutional Animal Care and Utilization Committee of Jilin University.

In vivo toxicity of CSHSs-Ar. To explore the systemic toxicity of CSHSs-Ar, ICR mice after adaptive feeding were randomly divided into 2 groups including control (no treatment) and skin exposure (500 μg/mL, 0.2 mL). After the above treatments, body weight and relative behavior of mice were recorded. A week later, whole blood was collected from the above 2 groups for hematological analysis and blood biochemical analysis. Meanwhile, skin and main organs were collected after mice were sacrificed and stained with hematoxylin and eosin (H&E) for further histological assessment.

Statistical analysis. Statistical analysis was carried out using OriginPro 2022 and GraphPad Prism 8. Significant differences were analyzed using the Student's *t*-test for two groups and one-way analysis of variance (ANOVA) for multiple group comparisons. All data in this study were expressed as mean ± standard deviation (SD) from at least 3 independent experiments. The p value less than 0.05 was considered statistically significant.

References

[1] M. Dolg, U. Wedig, H. Stoll, and H. Preuss. Energy-adjusted *ab initio* pseudopotentials for the first row transition elements. *The Journal of Chemical Physics* **1987**, *86,* 866.

[2] J. Perdew, K. Burke, and M. Ernzerhof. Generalized gradient approximation made simple. *Physical Review Letters* **1996**, *77,* 3865.

II. Supporting Figures and Tables

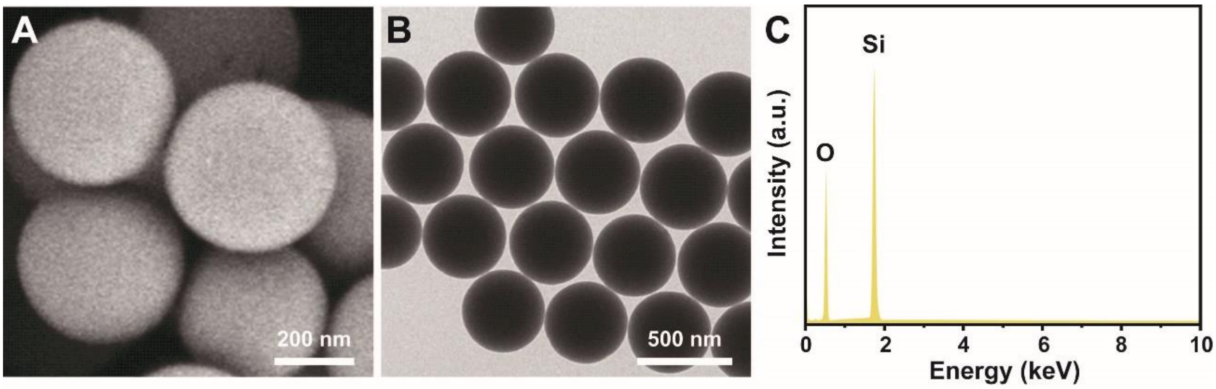

Figure S1. SEM image (A), TEM image (B), and EDS spectrum (C) of SiO_2 spheres.

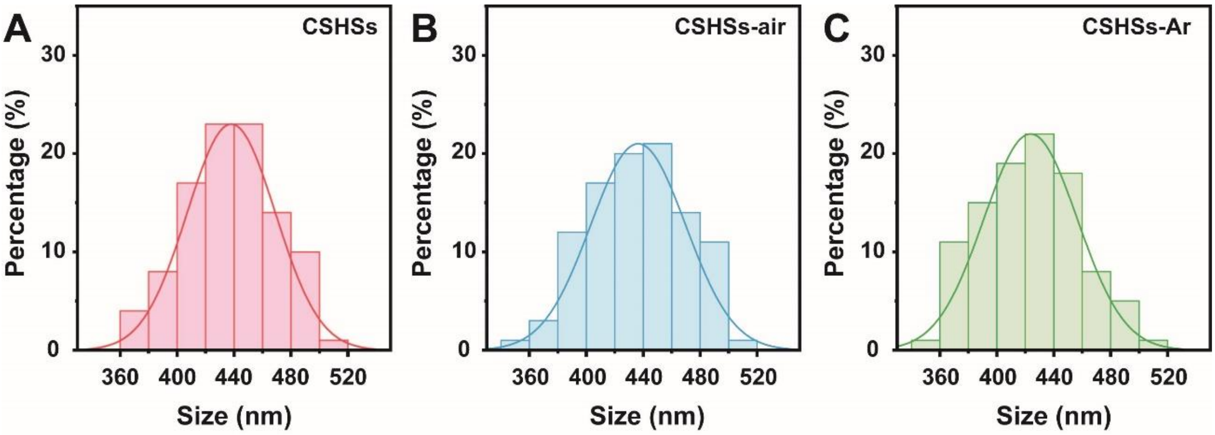

Figure S2. Size distributions of CSHSs (A), CSHSs-air (B), and CSHSs-Ar (C).

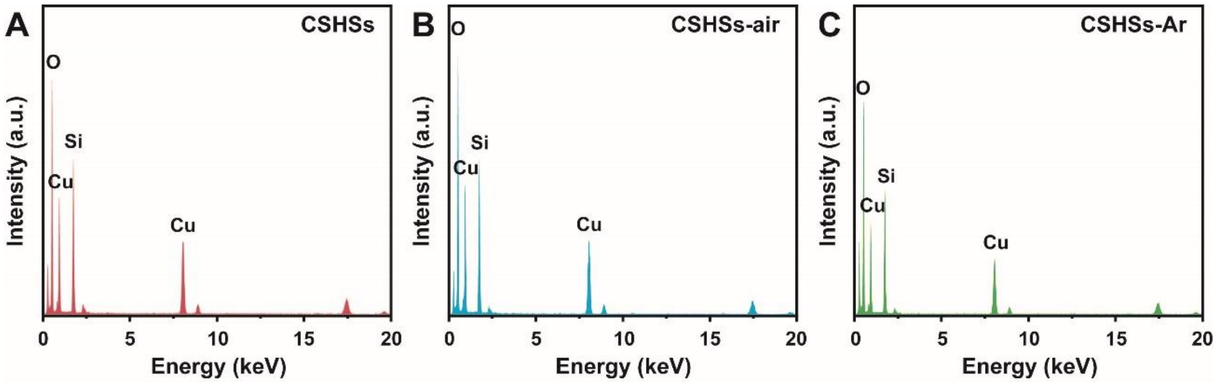

Figure S3. EDS spectra of CSHSs (A), CSHSs-air (B), and CSHSs-Ar (C).

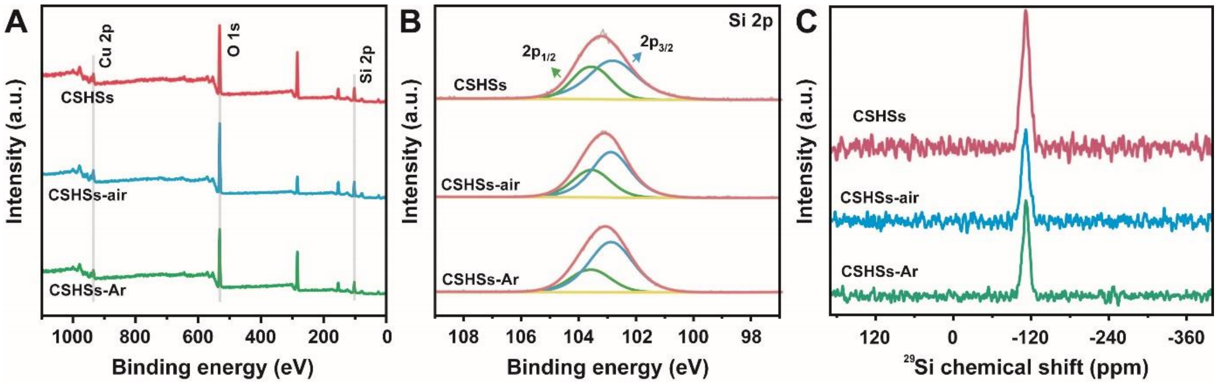

Figure S4. XPS survey spectra (A) and high-resolution XPS spectra of Si 2p (B) for CSHSs, CSHSs-air, and CSHSs-Ar. Solid-state ^{29}Si NMR spectra of CSHSs, CSHSs-air, and CSHSs-Ar (C).

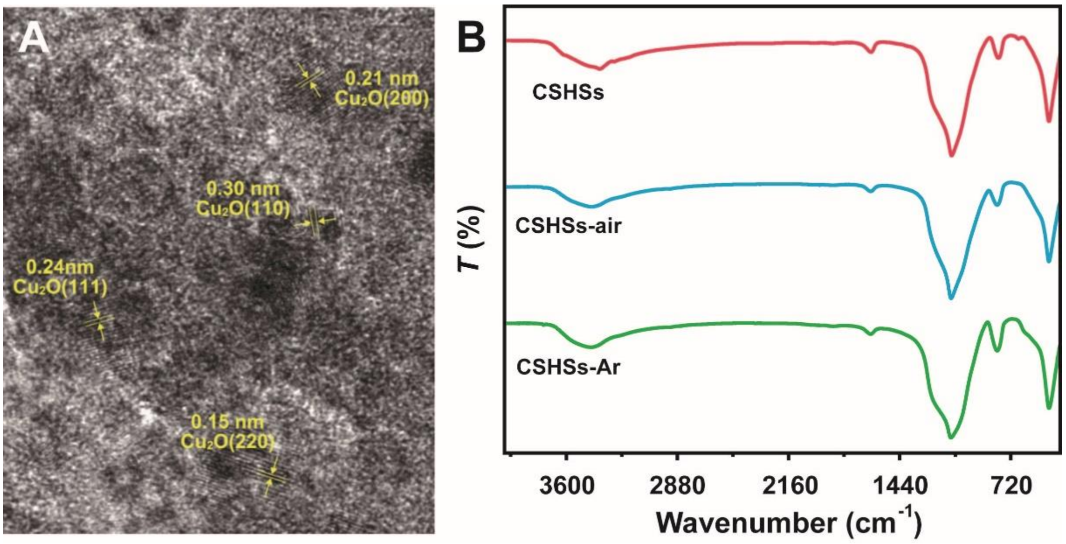

Figure S5. HR-TEM image of CSHSs-Ar (A). FT-IR spectra of CSHSs, CSHSs-air, and CSHSs-Ar (B).

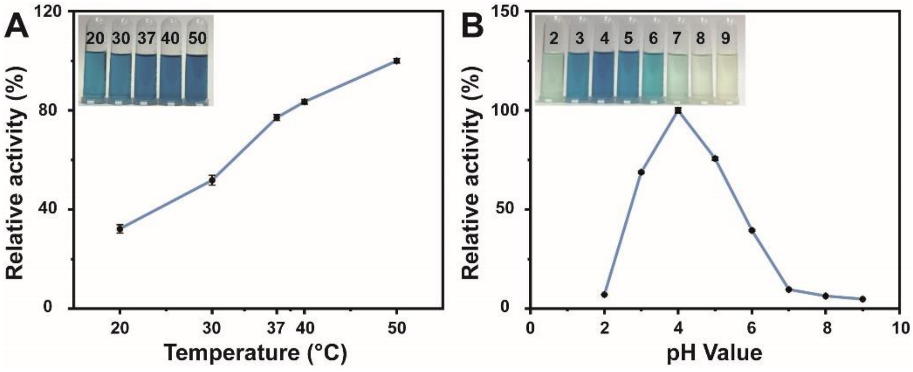

Figure S6. POD-like catalytic activity of CSHSs-Ar under different temperatures (A) and different pH values (B). In the typical experiment, the values of $OD_{652\ nm}$ of phosphate buffered saline (pH 4.0, 20 mM, 3 mL) containing TMB (1 mM), H_2O_2 (20 mM), and CSHSs-Ar (100 μg/mL) were recorded after the co-incubation. Data were presented as mean ± SD (n = 3).

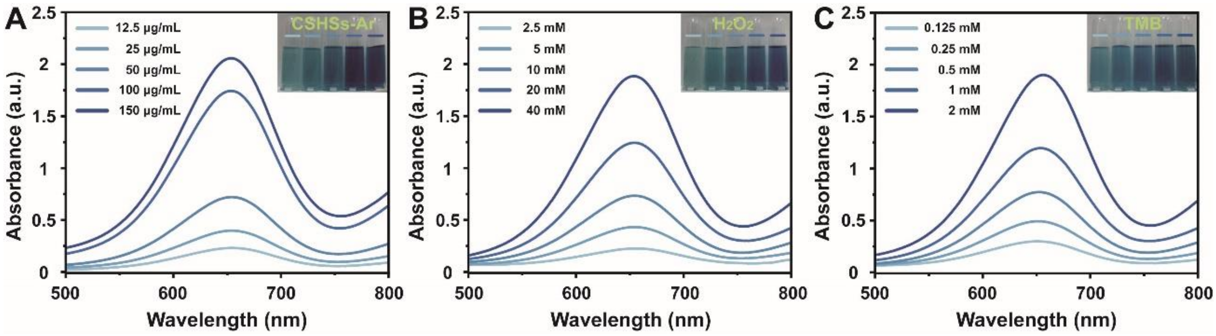

Figure S7. UV-vis absorption spectra of solutions containing different concentrations of CSHSs-Ar (A), H_2O_2 (B), and TMB (C). For A, the concentrations of TMB and H_2O_2 were 1 mM and 20 mM. For B, the concentrations of CSHSs-Ar and TMB were 100 μg/mL and 1 mM. For C, the concentrations of CSHSs-Ar and H_2O_2 were 100 μg/mL and 20 mM.

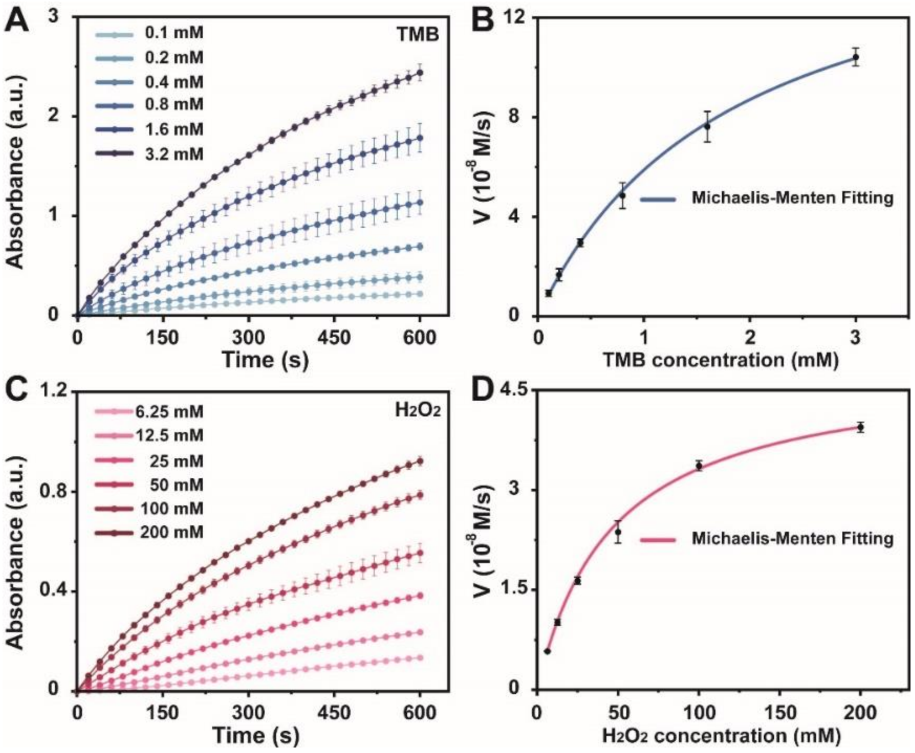

Figure S8. Time-dependent absorbance changes at 652 nm of solutions containing different concentrations of TMB (A) and H_2O_2 (C). Steady-state kinetics analysis of CSHSs-Ar for TMB (B) and H_2O_2 (D). Data were presented as mean ± SD (n = 3).

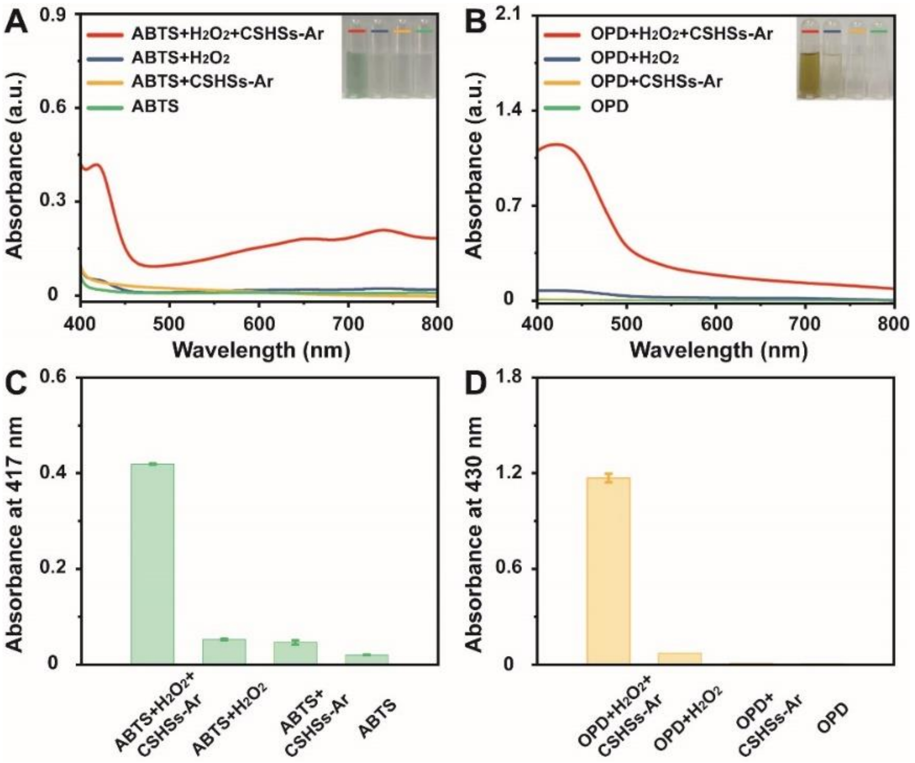

Figure S9. UV-vis absorption spectra and corresponding absorbance values of solutions containing ABTS (A, C) or OPD (B, D) receiving different treatments. Data in (C) and (D) were presented as mean ± SD (n = 3).

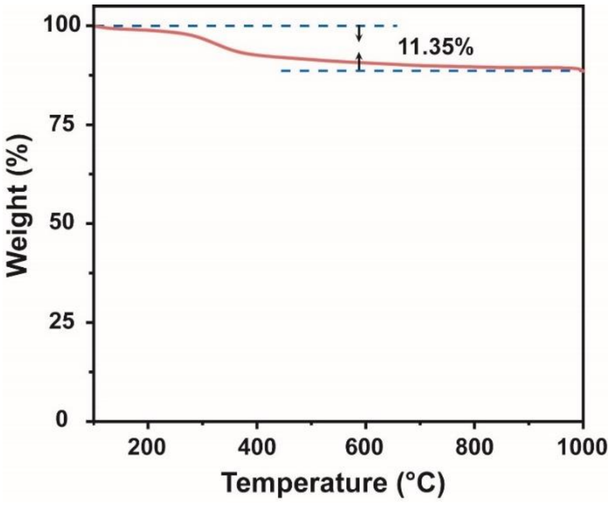

Figure S10. Thermogravimetric analysis (TGA) of CSHSs.

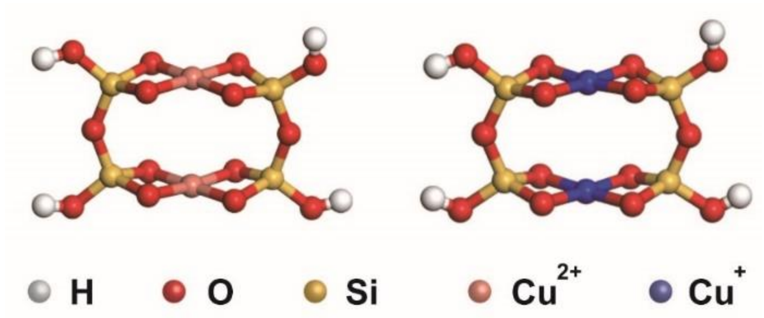

Figure S11. Geometry structure of copper with different valence states.

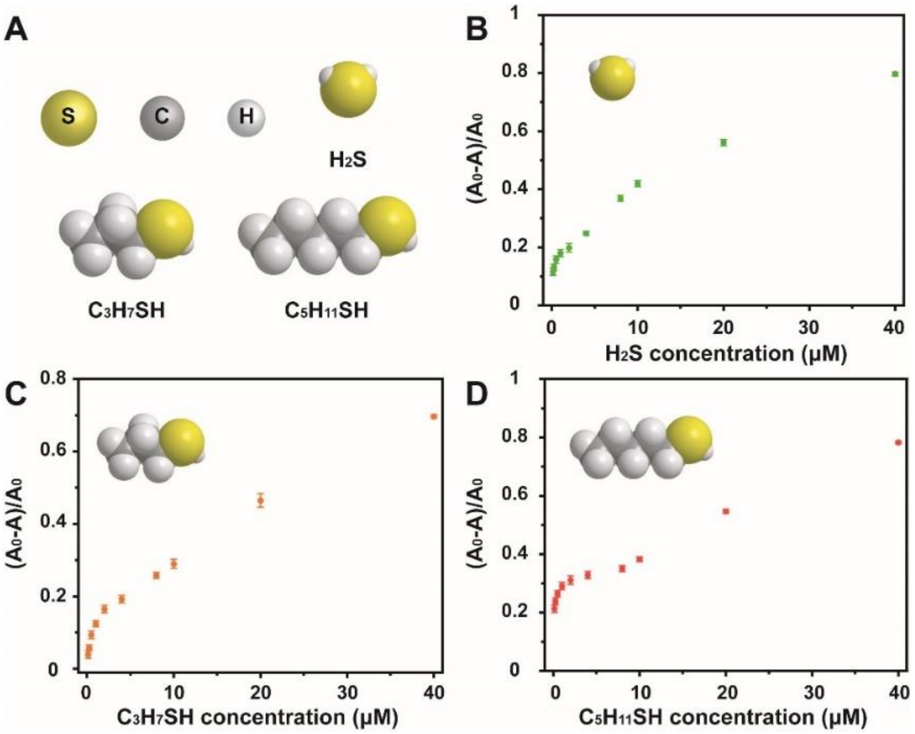

Figure S12. Chemical structures of H_2S, C_3H_7SH, and $C_5H_{11}SH$ (A). Concentration-dependent curves for H_2S (B), C_3H_7SH (C), and $C_5H_{11}SH$ (D) based on our thioalcohol detection system. Data in (B), (C), and (D) were presented as mean ± SD (n = 3).

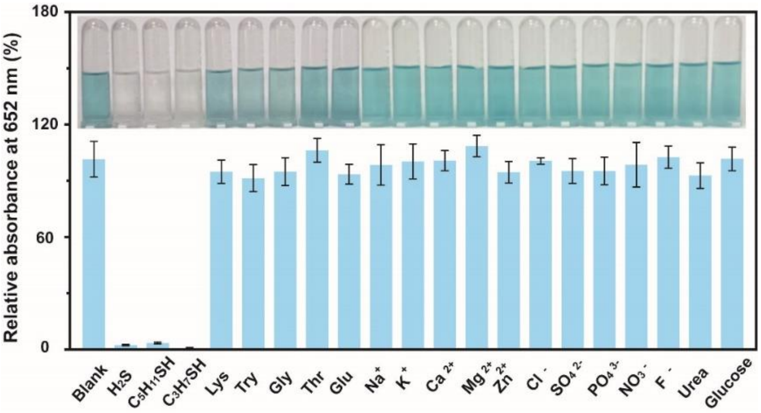

Figure S13. Normalized absorbance at 652 nm of CSHSs-Ar nanozyme-involved system containing H_2S, C_3H_7SH, $C_5H_{11}SH$, or the other interferences. Data were presented as mean ± SD (n = 3).

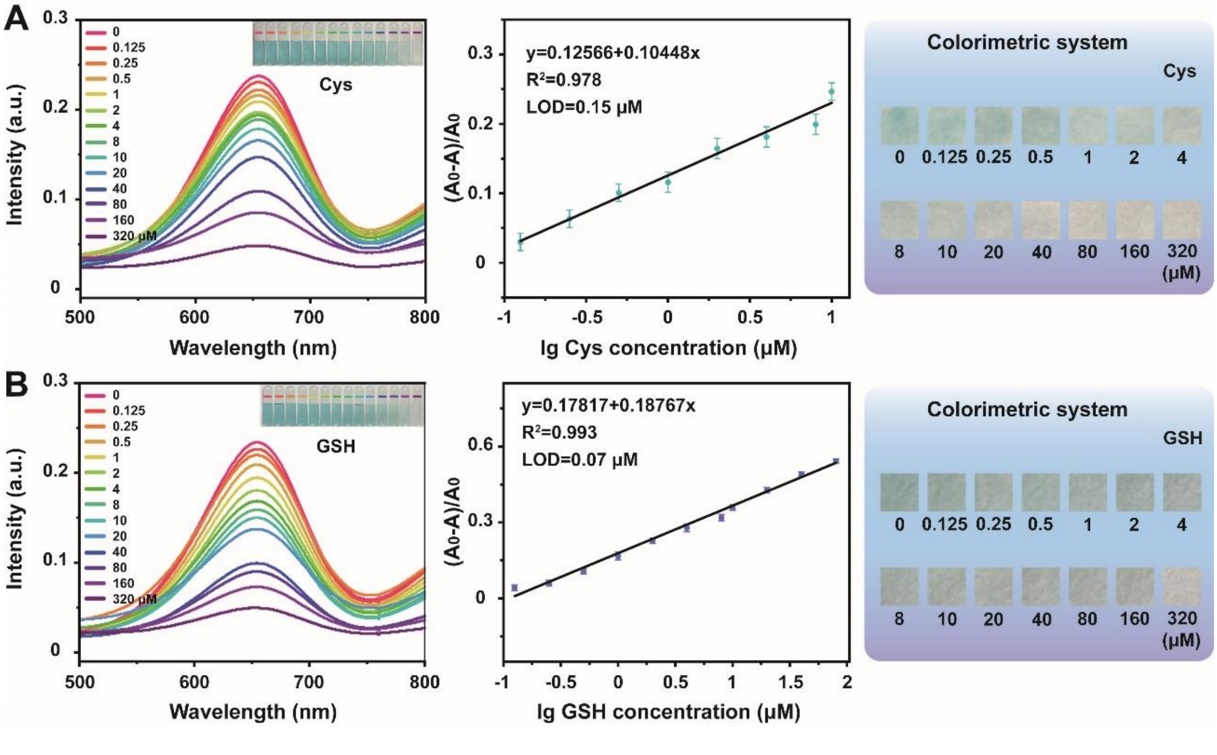

Figure S14. Concentration-dependent UV-vis absorbance spectra, linear curves with different concentration ranges, and corresponding discoloration photos of paper-based sensor for the detection of Cys (A) and GSH (B). Data were presented as mean ± SD (n = 3).

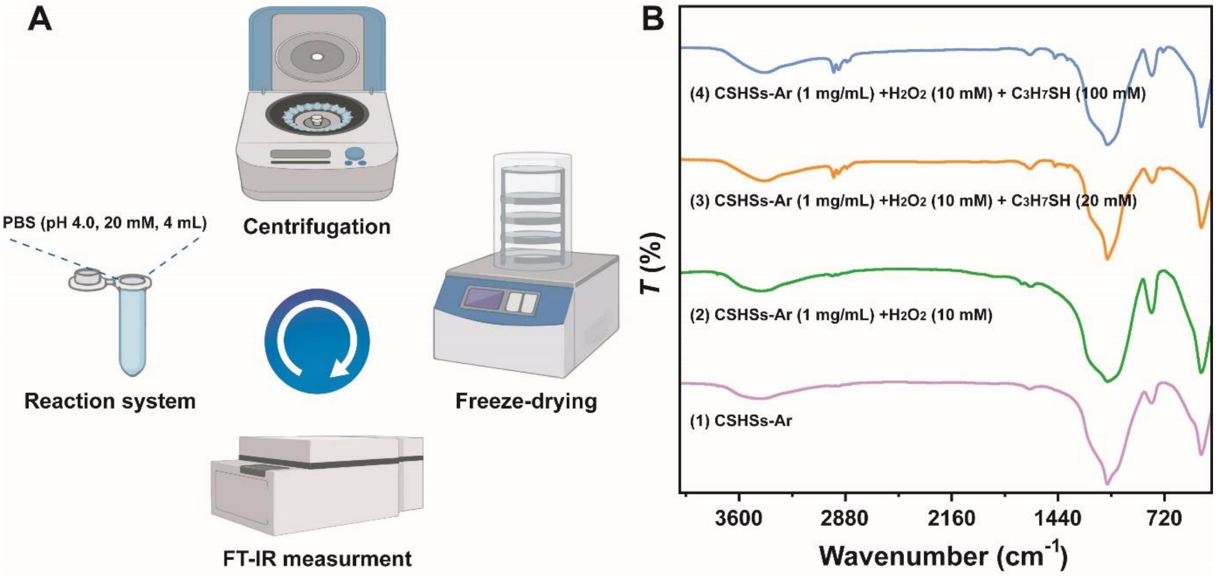

Figure S15. Schematic illustration for the preparation and FT-IR measurement of samples after different treatments (A). FT-IR spectra of the system containing CSHSs-Ar and H_2O_2 after the treatments of C_3H_7SH with different concentrations (B).

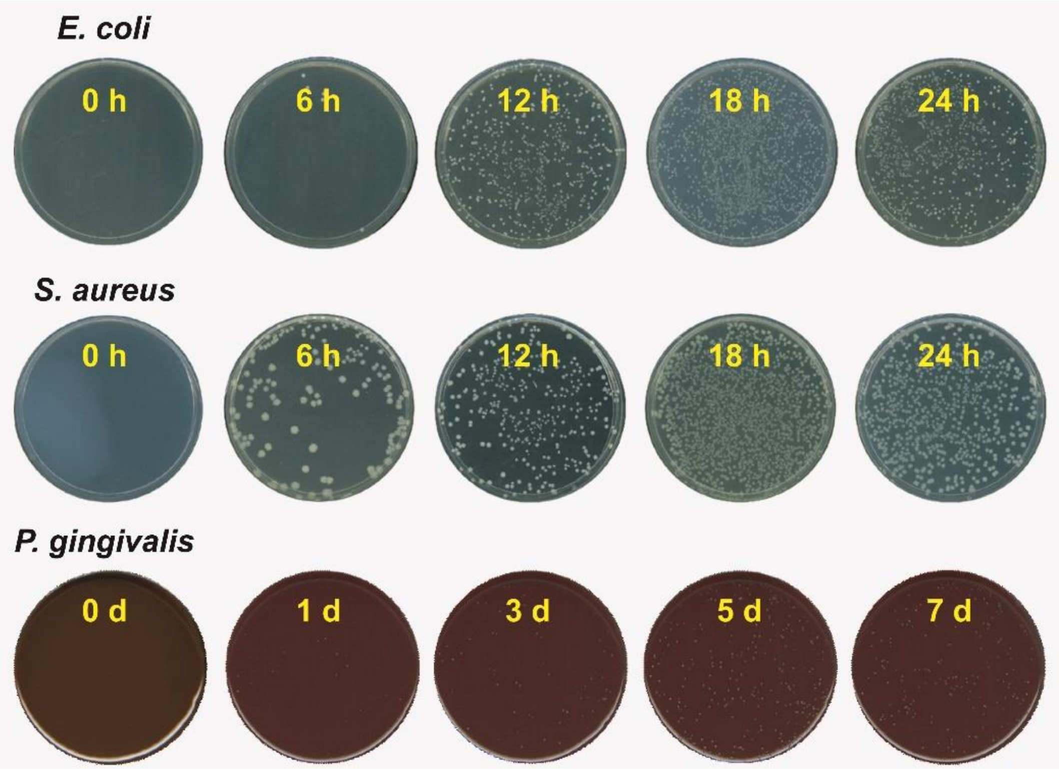

Figure S16. Enlarged image of Figure 5B.

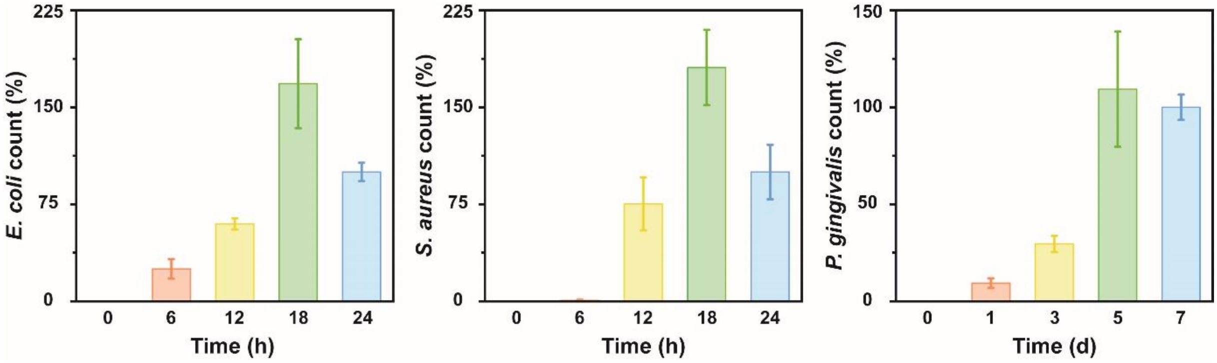

Figure S17. Relative bacterial numbers of *E. coli*, *S. aureus*, and *P. gingivalis* calculated by colony counting. The bacteria counts at the final detection were defined as 100%, respectively. Data were presented as mean ± SD (n = 3).

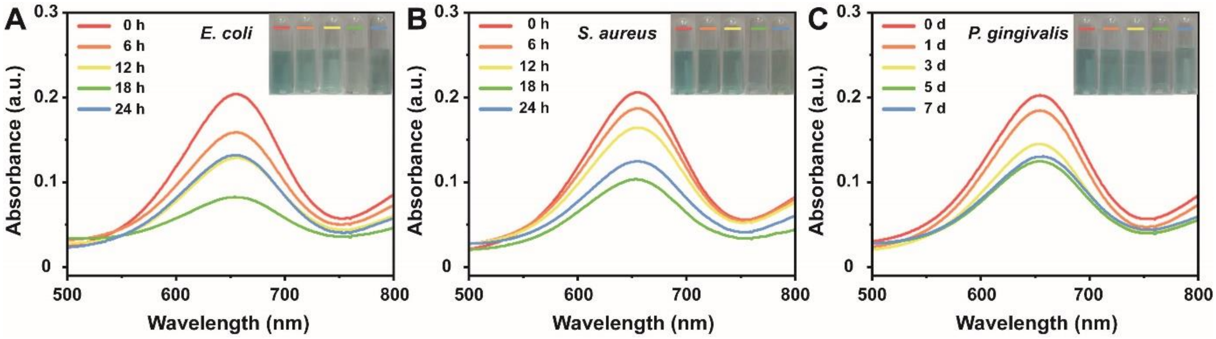

Figure S18. Co-incubation period-dependent UV-vis absorption spectra of solutions containing extracts from different bacterial culture mediums responding to *E. coli* (A), *S. aureus* (B), and *P. gingivalis* (C), respectively.

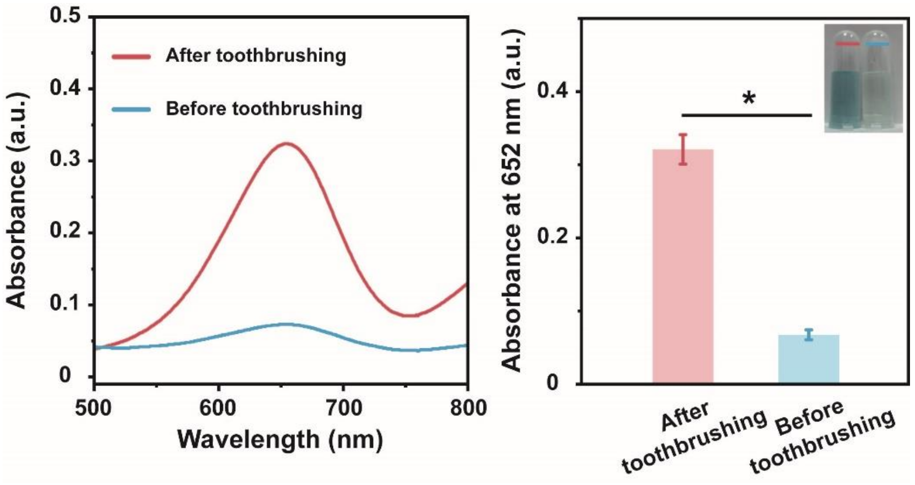

Figure S19. UV-vis absorption spectra of GCF samples from volunteers before and after toothbrushing based on our detection system, as well as corresponding quantitative results. Data were presented as mean ± SD (n = 3). Statistical significance was calculated using unpaired *t*-test. *p <0.05.

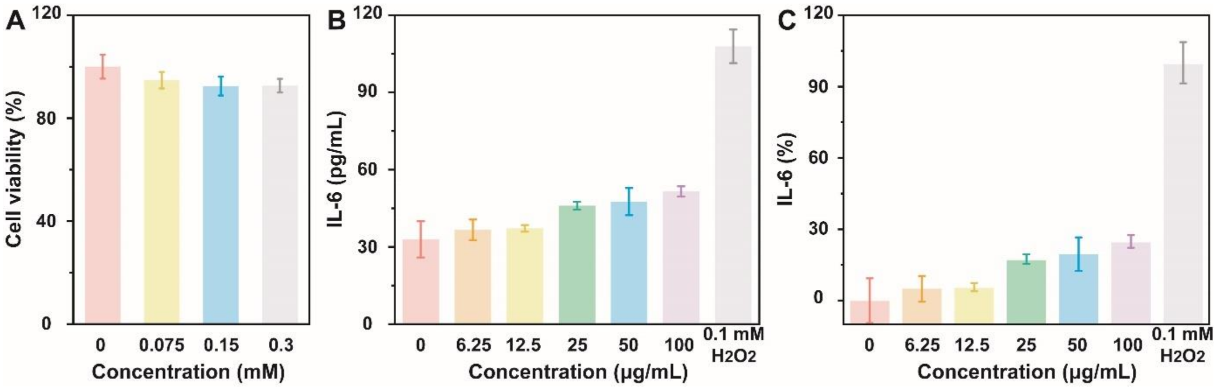

Figure S20. Viabilities of L929 cells after being co-incubated with different concentrations of H_2O_2 (A). The release amounts (B) and relative percentages (C) of IL-6 from L929 cells after different treatments. Data were presented as mean ± SD (n = 3).

Table S1. Summary of the content of copper with different valence states in various samples according to XPS analysis.

Samples	Percentage of Cu(I)	Percentage of Cu(II)
CSHSs	0	100
CSHSs-air	0	100
CSHSs-Ar	58.89	41.11

Table S2. Summary of the steady-state kinetic parameters of CSHSs-Ar, CSHSs, Fe_3O_4 nanozymes, and horseradish peroxidase (HRP).

Catalysts	Substrate	K_m (mM)	V_{max} (M/s)
CSHSs-Ar	H_2O_2	46.614	4.864×10^{-8}
	TMB	1.851	16.771×10^{-8}
CSHSs[1]	H_2O_2	97.31	3.47×10^{-8}
	TMB	2.492	7.625×10^{-8}
Fe_3O_4 nanozymes	H_2O_2	154	9.78×10^{-8}
	TMB	0.098	3.44×10^{-8}
HRP	H_2O_2	3.7	8.71×10^{-8}
	TMB	0.434	10.00×10^{-8}

[1] X. Liu, D. Luo, S. Dai, Y. Cai, T. Chen, X. Bao, M. Hu, and Z. Liu. Artificial bacteriophages for treating oral infectious disease via localized bacterial capture and enhanced catalytic sterilization. *Adv. Sci.* **2024**, *11*, 2400394.

Table S3. Detection performance of H_2S using previous Cu-based nanozyme-assisted sensors and CSHSs-Ar nanozyme-involved system.

Sensors	Liner range (μM)	LOD (μM)	Reference
CSHSs-Ar	0.125-4	0.15	Our study
MOF-818	0-400	0.8	[1]
GMP-Cu	0-220	0.67	[2]
Fe/Cu-N-C	0.09-6	0.03	[3]
Cu(I)-pTTCA	0.5-10	0.032	[4]
aCu(II)-CIFs	0.6-30	0.071	[5]

[1] K. Yu, M. Li, H. Chai, Q. Liu, X. Hai, M. Tian, L. Qu, T. Xu, G. Zhang, and X. Zhang. MOF-818 nanozyme-based colorimetric and electrochemical dual-mode smartphone sensing platform for in situ detection of H_2O_2 and H_2S released from living cells. *Chem. Eng. J.* **2023**, *451*, 138321.

[2] H. Huang, M. Li, M. Hao, L. Yu, and Y. Li. A novel selective detection method for sulfide in food systems based on the GMP-Cu nanozyme with laccase activity. *Talanta* **2021**, *235*, 122775.

[3] X. Chen, Y. Wang, M. Feng, D. Deng, X. Xie, C. Deng, K. Khattak, and X. Yang. Dual-active-site Fe/Cu single-atom nanozymes with multifunctional specific peroxidase-like properties for S^{2-} detection and dye degradation. *Chin. Chem. Lett.* **2023**, *34*, 107969.

[4] X. Gao, Z. Li, H. Wen, J. Zhao, M. Zhou, S. Yang, and J. Liu. Instant oxidase- and peroxidase-mimic activities of in-situ reductive coordinated Cu(I)-polytrithiocyanuric acid for H_2S colorimetric detection and antibacterial. *J. Hazard. Mater.* **2025**, *483*, 136722.

[5] Z. Ding, X. Gao, Y. Yang, H. Wei, S. Yang, and J. Liu. Amorphous copper(II)-cyanoimidazole frameworks as peroxidase mimics for hydrogen sulfide assay. *J. Colloid Interf. Sci.* **2023**, *652*, 1889.

Table S4. Detection performance of Cys using previous Cu-based nanozyme-assisted sensors and CSHSs-Ar nanozyme-involved system.

Sensors	Liner range (μM)	LOD (μM)	Reference
CSHSs-Ar	0.125-10	0.15	Our study
Cu-Asp CP	0-50	0.625	[1]
$CuMnO_2$	25-300	11.26	[2]
Cu@Au/Pt	0-400, 400-3000	4	[3]
Cu_2O NPs	0-10	0.81	[4]
CuBDC	0.75-150	0.67	[5]

[1] X. Sun, Z. Qin, J. Shen, X. Cao, B. Liu, and H. Wang. A Cu(II) coordination polymer-based catalytic sensing system for detecting cysteine and sulfur anions. *Anal. Methods* **2018**, *10*, 4387.

[2] Y. Chen, T. Chen, X. Wu, and G. Yang. $CuMnO_2$ nanoflakes as pH-switchable catalysts with multiple enzyme-like activities for cysteine detection. *Sensors Actuat. B-Chem.* **2019**, *279*, 374.

[3] C. Jiang, X. Wei, S. Bao, H. Tu, and W. Wang. Cu@Au(Ag)/Pt nanocomposite as peroxidase mimic and application of Cu@Au/Pt in colorimetric detection of glucose and L-cysteine. *RSC Adv.* **2019**, *9*, 41561.

[4] Y. Zhu, Z. Zhang, X. Song, and Y. Bu. A facile strategy for synthesis of porous Cu_2O nanospheres and application as nanozymes in colorimetric biosensing. *J. Mater. Chem. B* **2021**, *9*, 3533.

[5] X. Li, H. Zhou, F. Qi, X. Niu, X. Xu, F. Qiu, Y. He, J. Pan, and L. Ni. Three hidden talents in one framework: a terephthalic acid-coordinated cupric metal-organic framework with cascade cysteine oxidase- and peroxidase-mimicking activities and stimulus-responsive fluorescence for cysteine sensing. *J. Mater. Chem. B* **2018**, *6*, 6207.

Table S5. Detection performance of GSH using previous Cu-based nanozyme-assisted sensors and CSHSs-Ar nanozyme-involved system.

Sensors	Liner range (μM)	LOD (μM)	Reference
CSHSs-Ar	0.125-80	0.07	Our study
AuNPs@Cu-porphyrin MOF	1-100	0.1	[1]
CuS-PDA-Au	0.5-100	0.42	[2]
Fe-Cu MOFs	2-20	0.439	[3]
$Cu_{1.8}S$ NPs	500-1000	60	[4]
Cu/CuO-rGO	0.03-3.25	0.03	[5]

[1] K. Sun, C. Liu, Y. Cao, J. Zhu, J. Li, and Q. Huang. Colorimetric and SERS dual-mode detection of GSH in human serum based on AuNPs@Cu-porphyrin MOF nanozyme. *Anal. Chim. Acta* **2024**, *1304*, 342552.

[2] Y. Wang, Y. Liu, F. Ding, X. Zhu, L. Yang, P. Zou, H. Rao, Q. Zhao, and X. Wang. Colorimetric determination of glutathione in human serum and cell lines by exploiting the peroxidase-like activity of CuS-polydopamine-Au composite. *Anal. Bioanal. Chem.* **2018**, *410*, 4805.

[3] H. Li, H. Wu, J. Chen, Y. Su, P. Lin, W. Xiao, and D. Cao. Highly sensitive colorimetric detection of glutathione in human serum based on iron-copper metal-organic frameworks. *Langmuir* **2022**, *38*, 15559.

[4] H. Zou, T. Yang, J. Lan, and C. Huang. Use of the peroxidase mimetic activity of erythrocyte-like $Cu_{1.8}S$ nanoparticles in the colorimetric determination of glutathione. *Anal. Methods* **2017**, *9*, 841.

[5] P. Singh, P. Nath, R. Arun, S. Mandala, and N. Chanda. Novel synthesis of mixed Cu/CuO-reduced graphene oxide nanocomposite with enhanced peroxidase-like catalytic activity for easy detection of glutathione in solution and using paper strip. *RSC Adv.* **2016**, *6*, 92729.
